# Leveraging Climate Data for Dengue Forecasting in Ba Ria Vung Tau Province, Vietnam: An Advanced Machine Learning Approach

**DOI:** 10.3390/tropicalmed9100250

**Published:** 2024-10-21

**Authors:** Dang Anh Tuan

**Affiliations:** Faculty of Public Health, University of Medicine and Pharmacy at Ho Chi Minh City, Ho Chi Minh City 70000, Vietnam; tuanda222@gmail.com

**Keywords:** dengue fever, machine learning, climate forecasting, negative binomial regression, SARIMAX, XGBoost, LSTM, Ba Ria Vung Tau, Vietnam

## Abstract

Dengue fever is a persistent public health issue in tropical regions, including Vietnam, where climate variability plays a crucial role in disease transmission dynamics. This study focuses on developing climate-based machine learning models to forecast dengue outbreaks in Ba Ria Vung Tau (BRVT) province, Vietnam, using meteorological data from 2003 to 2022. We utilized four predictive models—Negative Binomial Regression (NBR), Seasonal AutoRegressive Integrated Moving Average with Exogenous Regressors (SARIMAX), Extreme Gradient Boosting (XGBoost) v2.0.3, and long short-term memory (LSTM)—to predict weekly dengue incidence. Key climate variables, including temperature, humidity, precipitation, and wind speed, were integrated into these models, with lagged variables included to capture delayed climatic effects on dengue transmission. The NBR model demonstrated the best performance in terms of predictive accuracy, achieving the lowest Mean Absolute Error (MAE), compared to other models. The inclusion of lagged climate variables significantly enhanced the model’s ability to predict dengue cases. Although effective in capturing seasonal trends, SARIMAX and LSTM models struggled with overfitting and failed to accurately predict short-term outbreaks. XGBoost exhibited moderate predictive power but was sensitive to overfitting, particularly without fine-tuning. Our findings confirm that climate-based machine learning models, particularly the NBR model, offer valuable tools for forecasting dengue outbreaks in BRVT. However, improving the models’ ability to predict short-term peaks remains a challenge. The integration of meteorological data into early warning systems is crucial for public health authorities to plan timely and effective interventions. This research contributes to the growing body of literature on climate-based disease forecasting and underscores the need for further model refinement to address the complexities of dengue transmission in highly endemic regions.

## 1. Introduction

Dengue fever (DF), a mosquito-borne viral infection, continues to pose a significant public health threat in tropical and subtropical regions worldwide, including Vietnam. The disease is transmitted predominantly by *Aedes aegypti* mosquitoes, whose breeding, survival, and transmission dynamics are strongly influenced by environmental factors such as temperature, precipitation, and humidity [1]. Ba Ria Vung Tau (BRVT), a coastal province in Southern Vietnam, regularly experiences outbreaks of dengue fever, making the development of accurate predictive models essential for the timely implementation of disease control measures [2]. Climate-based forecasting models, which incorporate meteorological data, have shown great promise in improving the accuracy of dengue outbreak predictions, thereby enabling more effective public health responses [3].

Several studies have confirmed the critical role of climatic factors in influencing the transmission of dengue. A study conducted in Southern China demonstrated that temperature, humidity, and rainfall are the most significant variables affecting the mosquito population and, consequently, the incidence of dengue fever [1]. Similarly, research in Queensland, Australia, using Bayesian spatial analysis, found a strong correlation between climatic variability and dengue transmission, with increased rainfall and higher temperatures significantly boosting the risk of outbreaks [3]. These findings highlight the importance of integrating climate data into predictive models to forecast dengue outbreaks effectively.

Machine learning (ML) and deep learning (DL) approaches have recently revolutionized the field of disease forecasting by providing more sophisticated tools for handling complex, non-linear relationships in time series data. Long short-term memory (LSTM) models, for instance, have proven highly effective in forecasting dengue outbreaks based on climate factors. A study conducted in China demonstrated that LSTM models outperformed traditional time-series models by reducing prediction error by as much as 24.91% when forecasting dengue cases one month in advance [4]. Furthermore, a dynamic ensemble approach combining weather and population susceptibility cycles was able to accurately predict dengue outbreaks in Brazil months ahead of time, demonstrating the significant potential of integrating meteorological and population data [5].

In Vietnam, probabilistic seasonal forecasting models have also proven valuable in predicting dengue outbreaks up to six months in advance. A superensemble model that integrates Earth observation data and seasonal climate forecasts has been shown to outperform baseline models, particularly in regions with semi-regular dengue transmission, such as Southern Vietnam. This model successfully predicted outbreak periods with an accuracy significantly higher than models that rely solely on historical incidence rates [2].

The integration of advanced machine learning techniques, including neural networks and hybrid models, has further refined dengue forecasting models. A study in Mexico employed a multi-stage machine learning approach to predict dengue outbreaks by analyzing the temporal dynamics of temperature and dengue data. This model outperformed several state-of-the-art forecasting methods and provided accurate predictions of dengue cases on an annual scale [6]. Another study in Malaysia demonstrated that support vector machine (SVM) models, when applied to meteorological data, achieved an accuracy rate of 70% in predicting dengue outbreaks, highlighting the potential of machine learning techniques in dengue forecasting [7].

Given the success of climate-based predictive models in forecasting dengue outbreaks, this study aims to develop a robust model for predicting dengue outbreaks in BRVT, using meteorological data. By leveraging advanced machine learning techniques and incorporating key climatic variables such as temperature, humidity, wind speed, rainfall, and some additional variables such as surface pressure, wind direction, and sea surface temperature (SST), this model seeks to provide accurate, early warnings of potential dengue outbreaks. The findings of this study will contribute to the growing body of research focused on climate-based early warning systems for vector-borne diseases and provide a practical tool for public health officials in dengue-endemic regions.

## 2. Materials and Methods

### 2.1. Study Area

This study was conducted in Ba Ria Vung Tau (BRVT), a coastal province located in Southern Vietnam (approximately between latitudes 10°20′ N and 10°40′ N, and longitudes 107°00′ E and 107°30′ E), which is highly vulnerable to dengue outbreaks due to its tropical monsoon climate. The province spans an area of about 1982 square kilometers, with a population exceeding 1.1 million as of the 2020 census. The region experiences distinct wet and dry seasons, with the rainy season typically occurring from May to October, characterized by high humidity and heavy rainfall, providing optimal breeding conditions for *Aedes aegypti* mosquitoes, the primary vector for dengue transmission [8].

BRVT’s geographic diversity, featuring urban centers, rural farmlands, and coastal zones, creates a range of microclimates that influence local weather patterns, including temperature variability and humidity levels, both of which are known to impact mosquito breeding and dengue transmission dynamics [9]. Additionally, BRVT’s varying elevations and coastal proximity further shape its climate, heightening the region’s vulnerability to dengue outbreaks.

The choice of BRVT also considers the climatic differences between Northern and Southern Vietnam. While Northern Vietnam, such as Hanoi, experiences significant seasonal temperature fluctuations, Southern Vietnam, including BRVT, maintains more stable temperatures throughout the year but with high humidity and intense rainfall during the rainy season. These conditions make Southern Vietnam more prone to continuous dengue transmission. The consistent occurrence of dengue cases in BRVT in recent years, coupled with its exposure to climatic fluctuations, makes it an ideal location for research focused on climate-based dengue forecasting. This setting allows for the development of predictive models tailored to the specific environmental conditions of Southern Vietnam, where dengue remains a significant public health concern.

### 2.2. Data Collection

Epidemiological Data: Weekly data on confirmed dengue fever cases reported during the study period from 1 January 2003 to 31 December 2022 were obtained from the Center for Disease Control of Ba Ria Vung Tau Province, Vietnam.

Laboratory Confirmation of Dengue Cases: In this study, the dengue cases used for analysis were laboratory-confirmed cases, as recorded by the Center for Disease Control of Ba Ria Vung Tau Province. The confirmation followed national guidelines for dengue surveillance in Vietnam, which involve diagnostic testing using enzyme-linked immunosorbent assay (ELISA) to detect dengue-specific IgM antibodies or NS1 antigen. In some cases, a reverse transcription–polymerase chain reaction (RT-PCR) was employed for detecting viral RNA, which provided a more specific diagnosis. These methods help differentiate dengue virus infections from other arboviruses, such as Chikungunya and Zika, which present with similar clinical symptoms.

Climate Data: The climate parameters presented in Table 1 were used in our model. These variables were selected based on previous studies and their assumed relationship with dengue fever. The data were collected from daily datasets with original resolution from NASA/POWER CERES/MERRA2, spanning from 1 January 2003 to 31 December 2022. Location coordinates: Latitude 10.5417, Longitude 107.243. Altitude from MERRA-2: Average for a region of 0.5 × 0.625 degrees latitude/longitude = 35.52 m. To assess the accuracy of the models in determining the relationship between these variables and dengue cases, we used the number of dengue cases as a benchmark for both regression and classification tasks.

### 2.3. Data Preprocessing

#### 2.3.1. Missing Data Imputation

Due to occasional gaps in the meteorological datasets, two imputation techniques were employed:

Linear Interpolation: Missing values for continuous variables such as temperature and humidity were filled using linear interpolation to maintain the temporal continuity of the data [1].

K-Nearest Neighbors (KNN) Imputation: For more substantial gaps, the KNN imputation technique was used. This method estimates missing values based on the similarity to neighboring data points in the feature space, ensuring robust predictions [10].

#### 2.3.2. Feature Engineering

Several new features were engineered to enhance the predictive power of the model:

Lagged Variables: Climate variables (temperature, precipitation, humidity) were shifted by 2 to 20 weeks to capture the delayed effects of weather conditions on dengue transmission. This accounts for the lifecycle of mosquitoes and the incubation period of the virus [11].

Rolling Averages: To capture the cumulative effects of climate conditions, rolling averages were computed for key variables across different time windows (ranging from 1 to 20 weeks), helping to model sustained climate effects on dengue outbreaks.

Seasonal and Monthly Indicators: Categorical variables representing the season and month were included to account for the strong seasonality in dengue incidence, with dummy variables for each season (spring, summer, autumn, and winter).

### 2.4. Feature Scaling

To ensure that all features contributed equally to the model, standardization was applied to the dataset. Using StandardScaler from scikit-learn, all continuous features were transformed to have a mean of 0 and a standard deviation of 1. This process prevents features with larger numerical ranges from dominating the model training process [12].

### 2.5. Feature Selection

A correlation matrix was computed to examine the relationships between the climate variables and the target variable, total dengue cases. The features showing the highest correlation with dengue cases were selected for further analysis. These lagged features were selected as they demonstrated the strongest correlations with the target variable (total dengue cases).

### 2.6. Data Splitting

The dataset, which includes dengue fever cases and weather variables from week 01 of 2003 to week 53 of 2022, was used for this study. Before analysis, feature engineering was performed to create new features from the variables or to transform the original data into more informative formats. Two datasets were considered: the first dataset consisted of raw data, including weather parameters (Table 1) and time variables, and is referred to as DS1. The second dataset contained time variables and the number of dengue cases, referred to as DS2. To capture the delayed effects of climatic factors on dengue transmission, cross-correlation analysis was conducted to determine the appropriate lag time for each meteorological variable, ranging from 0 to 12 months. Similarly, autocorrelation analysis (ACF) was conducted to evaluate the impact of past dengue cases on current cases.

Before conducting the analysis, the datasets were split into training and test sets. The training data consisted of observations from week 01 of 2003 to week 17 of 2017 for both DS1 and DS2. Likewise, the test data covered the period from week 18 of 2017 to week 53 of 2022 for both DS1 and DS2. The training dataset was used to build the models, while the test dataset was used to validate the models.

### 2.7. Data Modeling

**Figure 1:**
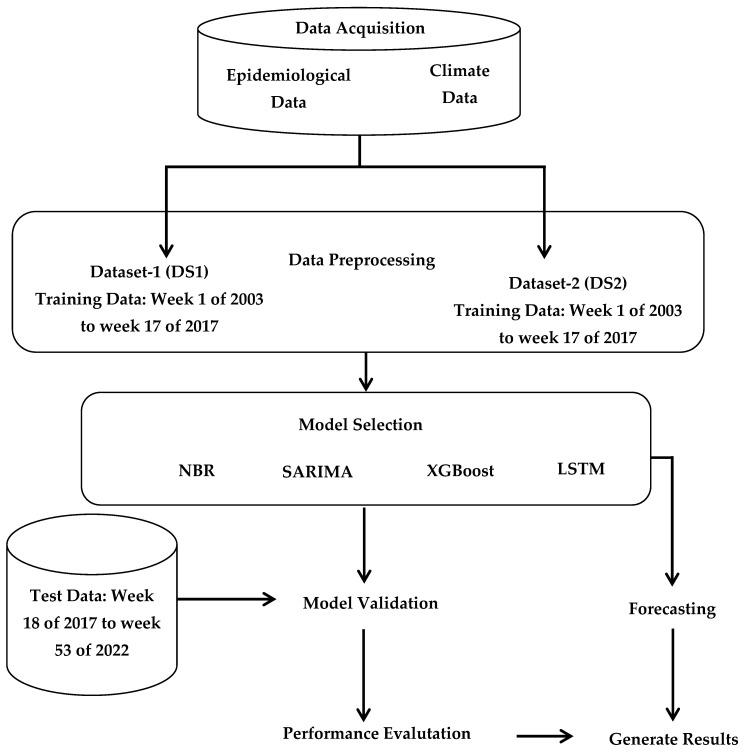
Overall process of DF case prediction study.

In this study, statistical, machine learning, and deep learning models such as Negative Binomial Regression (NBR), Seasonal AutoRegressive Integrated Moving Average with Exogenous Regressors (SARIMAX), Extreme Gradient Boosting (XGBoost) Regression, and long short-term memory (LSTM) were deployed to predict dengue cases in BRVT. The overall framework of the study and step-by-step modeling workflow is illustrated in Figure 1. Dengue cases were treated as the dependent or response variable, while the climatic variables and their respective lags were used as independent variables.

#### 2.7.1. Negative Binomial Regression (NBR)

Negative Binomial Regression (NBR) was selected to predict dengue case counts due to the overdispersed nature of the data, where the variance substantially exceeded the mean. NBR is preferable to Poisson regression in such cases, as it introduces a dispersion parameter to account for overdispersion [13].

The NBR model relates the expected number of dengue cases ( $\mu_{i}$ ) to the predictor variables ( $X_{i}$ ) through a log link function:

$$\log\left(\mu_{i}\right)=\beta_{0}+\sum_{k=1}^{p}\beta_{k}X_{ik}$$

where $\mu_{i}$ is the expected count of dengue cases, $X_{ik}$ are the predictor variables (e.g., temperature, humidity, lagged climatic variables), $\beta_{k}$ are the regression coefficients, and $\beta_{0}$ is the intercept.

NBR introduces a dispersion parameter *α* to account for overdispersion, which modifies the variance function:

$$Var\left(Y_{i}\right)=\mu_{i}+\alpha \mu_{i}^{2}$$

This variance function allows the model to adjust for the extra variability observed in dengue case counts [13].

Model fitting was performed using maximum likelihood estimation (MLE). The dispersion parameter *α* was tuned via cross-validation. A grid search was conducted over a range of *α* values (0.001 to 2.0) to optimize model performance based on the Mean Absolute Error (MAE) on the test set.

The steps for model tuning included the following: (i) Train–test split—Data were used for training (80%), and data were used for testing (20%) to evaluate the model’s predictive performance; (ii) Cross-validation—A rolling window time-series cross-validation approach was employed to prevent data leakage and ensure temporal consistency [14]; (iii) Hyperparameter Optimization—GridSearchCV was used to identify the best-fitting model by minimizing MAE [15].

#### 2.7.2. Seasonal AutoRegressive Integrated Moving Average with eXogenous Regressors (SARIMAX)

The SARIMAX model was selected for dengue case prediction due to its ability to model both temporal dynamics and the influence of exogenous variables, such as climate factors. SARIMAX extends the classical ARIMA (Autoregressive Integrated Moving Average) model by incorporating seasonal components and exogenous variables, which makes it particularly well-suited for modeling seasonal data with external influences.

The SARIMAX model is defined by the following components:

AR (Autoregressive) component: This component captures the relationship between an observation and its previous values.

I (Integrated) component: This accounts for differencing the series to make it stationary, reducing the impact of trends.

MA (Moving Average) component: This models the error terms as a linear combination of past error terms.

Seasonality: SARIMAX accounts for seasonality through seasonal AR, I, and MA terms.

The general SARIMAX model is written as:

$$Y_{t}=\sum_{i=1}^{p}\emptyset_{i}Y_{t-i}+\sum_{j=1}^{q}\theta_{j}\epsilon_{t-j}+X_{t}\beta +\epsilon_{t}$$

where $Y_{t}$ is the dengue case count at time *t*, $\emptyset_{i}$ are the autoregressive coefficients, $\theta_{j}$ are the moving average coefficients, $\epsilon_{t}$ is the error term (white noise), $X_{t}$ represents the exogenous variables (e.g., climate variables such as temperature, humidity, precipitation), and *β* is the vector of coefficients associated with the exogenous variables.

For the seasonal component, the model is extended by introducing seasonal autoregressive (SAR), seasonal moving average (SMA), and seasonal differencing terms, denoted as *P*, *D*, and *Q*, respectively. The seasonal component is added with periodicity *s*, representing the number of time steps in a season.

The full SARIMAX model is typically written as follows:

$${SARIMA(p,d,q)(P,D,Q)}_{s}$$

where *p*, *d*, and *q* represent the non-seasonal parameters (autoregressive, differencing, and moving average), *P*, *D*, and *Q* are the seasonal counterparts, and *s* is the length of the seasonal cycle.

The exogenous variables ( $X_{t}$ ) included in the SARIMAX model were climate factors such as temperature, humidity, and precipitation, along with their lagged versions. These variables were selected based on their known impact on dengue transmission and mosquito population dynamics [16]. Lagged versions of these variables (1 to 4 weeks) were included to capture delayed climatic effects on dengue incidence.

The SARIMAX model was tuned using a grid search approach to identify the optimal values for the parameters *p*, *d*, *q*, *P*, *D*, *Q*, and *s*. The following steps were undertaken: (i) Train–test split—The dataset was split into training and testing sets. The training set was used to fit the model, while the test set was used for out-of-sample evaluation. (ii) Grid Search—A grid search was conducted over a range of possible values for the hyperparameters *p*, *d*, *q*, *P*, *D*, and *Q*, along with seasonal periodicity *s* = 52, to find the optimal combination that minimized the Akaike Information Criterion (AIC) [17]. (iii) Cross-validation—Time series cross-validation using a rolling window approach was implemented to ensure that temporal dependencies were preserved and to prevent data leakage during model evaluation [14].

#### 2.7.3. Extreme Gradient Boosting (XGBoost) Regression

XGBoost (Extreme Gradient Boosting) was selected for this study due to its efficiency and performance in handling large, complex datasets with non-linear relationships. XGBoost is a supervised machine learning algorithm that builds an ensemble of decision trees using a boosting technique to improve prediction accuracy. The algorithm works by iteratively adding weak learners (trees) to minimize the residual errors from previous iterations.

XGBoost operates by minimizing a loss function, typically the Mean Squared Error (MSE) for regression tasks, along with a regularization term to control model complexity and prevent overfitting. The objective function is given by:

$$Obj\left(\theta \right)=\sum_{i=1}^{n}L(y_{i},{\hat{y}}_{i})+\sum_{k=1}^{K}\Omega (f_{k})$$

where $L(y_{i},{\hat{y}}_{i})$ represents the loss function (e.g., squared error) between the actual values $y_{i}$ and predicted values ${\hat{y}}_{i}$ , $\Omega (f_{k})$ is the regularization term applied to each decision tree $f_{k}$ , which penalizes the complexity of the model to avoid overfitting [18].

The regularization term is defined as follows:

$$\Omega \left(f\right)=\gamma T+\frac{1}{2}\lambda \sum_{j=1}^{T}w_{j}^{2}$$

where *T* is the number of leaves in the tree, $w_{j}$ are the leaf weights, and *γ* and *λ* are regularization hyperparameters that control the trade-off between model complexity and training performance.

The XGBoost model includes several key hyperparameters that were tuned using grid search to optimize the model’s performance: (i) Learning Rate (*η*)—Controls the step size at each iteration of boosting. A lower learning rate requires more trees to achieve convergence but typically leads to better generalization. (ii) Max Depth—Controls the maximum depth of each decision tree. A deeper tree captures more complex patterns but increases the risk of overfitting. (iii) Subsample—Fraction of the training data used to build each tree. Subsampling helps prevent overfitting by introducing randomness into the training process. (iv) Gamma (*γ*)—Minimum loss reduction required to make a further partition on a leaf node of the tree. (v) Min Child Weight—Minimum sum of instance weights needed in a child node.

The hyperparameters were optimized using GridSearchCV with 5-fold cross-validation to ensure robustness. The combination of hyperparameters that minimized the Root Mean Square Error (RMSE) was selected for the final model [18].

#### 2.7.4. Long Short-Term Memory (LSTM)

Long short-term memory (LSTM) is the most effective approach for time series forecasting [19], where classical linear methods are difficult to use for multivariate forecasting issues. Among various machine learning methods, the recurrent neural network (RNN) uses feedback loops; subsequently, the network can learn the sequence of information. However, standard RNN fails to remember long-term information; hence, the present study proposes an LSTM network for dengue prediction.

LSTM is the recurrent neural network (RNN) architecture, and it was designed by Hochreiter and Schmidhuber to address the vanishing and exploding gradient problems of traditional RNNs [20]. These vanishing and gradient issues hinder the model’s accuracy. The LSTM consists of different blocks called memory cells. Memory block contains memory cells and gates. Memory cells are able to remember and manipulate the temporal state of the memory or network by self-connections, and the process of memory or information is controlled through three gates. The first gate is called the “Forget” gate ( $f_{j}$ ), which is responsible for removing information. The second gate is “Input” gate ( $i_{j}$ ), which is responsible for the addition of information to the cell state. The final gate is the “Output” gate ( $o_{j}$ ), which selects the useful information from the current cell and shows it as an output [21]. The forget gate, input gate, and output gate of LSTM are described by [20] using the following notations mentioned below:

$$f_{j}=\sigma (w_{f}\cdot \left[h_{j-1},x_{j}\right]+b_{f})\;i_{j}=\sigma (w_{i}\cdot \left[h_{j-1},x_{j}\right]+b_{i})\;o_{j}=\sigma \left(w_{o}\cdot \left[h_{j-1},x_{j}\right]+b_{o}\right)\;\hat{C}=\tan h\left(w_{c}\cdot \left[h_{j-1},x_{j}\right]+b_{c}\right)\;C_{j}=f_{j}*C_{j-1}+i_{j}*\hat{C}\;h_{j}=o_{j}*\tan h\left(C_{j}\right)$$

where f,i, and o are the forget gate, input gate, and output gate, respectively. The input gate is used to decide the information to be stored in the cell, the forget gate is used to decide what kind of information is to be dropped from the cell, and the output gate is used to decide the output from the cell. σ represents the logistic sigmoid function. w,b , and h are weights, biases, and values of hidden layers, respectively. $\hat{C}$ and C are vectors of new candidate values and cell states, respectively.

### 2.8. Predictive Performance and Model Validation

This study employed a comprehensive approach to evaluate the predictive performance of models developed for dengue case forecasting. Rigorous validation processes were implemented to ensure robustness, accuracy, and generalizability. Several error metrics, cross-validation techniques, and hyperparameter tuning strategies were used to optimize model performance.

#### 2.8.1. Evaluation Metrics

The predictive models were evaluated using key metrics designed to measure accuracy and error distribution:

Mean Absolute Error (MAE): MAE is calculated as the mean of the absolute differences between predicted and actual values, providing a direct measure of prediction accuracy. The formula for MAE is the following:

$$MAE=\frac{1}{n}\sum_{i=1}^{n}\left|y_{i}-{\hat{y}}_{i}\right|$$

where $y_{i}$ represents the actual values, ${\hat{y}}_{i}$ the predicted values, and *n* the number of observations [22].

Root Mean Square Error (RMSE): RMSE was used to give more weight to larger errors and is expressed as follows:

$$RMSE=\sqrt{\frac{1}{n}\sum_{i=1}^{n}{\left(y_{i}-{\hat{y}}_{i}\right)}^{2}}$$

RMSE is particularly sensitive to outliers and thus provides insight into the error distribution [23].

For time-series models such as SARIMAX, the Akaike Information Criterion (AIC) was employed to evaluate model complexity relative to goodness of fit. A lower AIC indicates a better fit when comparing models of different complexity [17]. The AIC formula is as follows:

$$AIC=2k-2\ln\left(\hat{L}\right)$$

where k is the number of parameters in the model, and $\hat{L}$ is the maximized likelihood.

#### 2.8.2. Model Validation Approach

The dengue dataset, spanning from week 1 of 2003 to week 17 of 2017, was split into two subsets:

Training Set (80%): The initial 80% of the data were reserved for training the models.

Testing Set (20%): The final 20% of the data were used to evaluate the models’ out-of-sample predictive performance.

For time-series forecasting models, including SARIMAX and LSTM, temporal integrity was preserved by ensuring that future observations were not included in the training phase, thereby replicating real-world forecasting conditions [24].

#### 2.8.3. Cross-Validation Strategy

To prevent data leakage and ensure model robustness, we employed TimeSeriesSplit cross-validation for machine learning models like XGBoost. This method expands the training set sequentially while moving the validation set forward in time, preserving the temporal structure of the data [14].

Hyperparameter optimization was performed using GridSearchCV, which systematically explored the hyperparameter space to find the optimal combination of parameters. This search was combined with 5-fold cross-validation to ensure robustness and generalizability [18].

#### 2.8.4. Overfitting and Generalization

To monitor overfitting, the models’ performance on both training and test datasets was compared:

Negative Binomial Regression: The model demonstrated consistent performance across training and testing data, with similar MAE scores indicating strong generalization capability. Negative Binomial Regression is particularly effective for count data with overdispersion, where the variance exceeds the mean [13]. The formula for Negative Binomial Regression is as follows:

$$\log\left(\mu_{i}\right)=X_{i}\beta$$

where $\mu_{i}$ represents the expected number of cases, $X_{i}$ the predictor variables, and β the coefficients.

SARIMAX: The baseline SARIMAX model exhibited overfitting, with significantly lower MAE and RMSE on the training set compared to the test set. The inclusion of exogenous variables (e.g., climate factors) in the multivariate SARIMAX model improved generalization and reduced overfitting [23].

XGBoost: Initially, the XGBoost model captured seasonal patterns but struggled with the prediction of outbreak peaks. The introduction of lagged climate variables significantly improved model performance, reducing generalization error and enhancing predictive accuracy [18].

#### 2.8.5. Model Refinement and Iteration

Several iterations were conducted to optimize the models:

Negative Binomial Regression: Incorporating time-shifted climate variables improved the model’s ability to predict weekly dengue case counts more accurately.

SARIMAX: The addition of lagged exogenous variables helped improve the model’s accuracy in capturing both seasonal trends and outbreak peaks.

XGBoost: The introduction of lagged variables in XGBoost further enhanced its ability to generalize across both training and testing datasets, resulting in improved MAE and RMSE scores.

#### 2.8.6. Final Model Selection

After iterative optimization, the final models were selected based on their performance in both training and testing datasets. These models were then retrained on the full dataset to maximize their predictive capabilities for future dengue cases. Feature importance analysis was also performed to identify the most significant predictors of dengue outbreaks [18].

### 2.9. Statistical Analysis

Once the final dataset was prepared, various machine learning models were trained to predict weekly dengue cases based on the selected climate features. The models’ performance was evaluated using metrics such as Root Mean Square Error (RMSE) and Mean Absolute Error (MAE). The analysis was performed using Python 3.12.4, leveraging libraries such as Pandas, NumPy, scikit-learn, and Seaborn for data processing, modeling, and visualization.

### 2.10. Ethical Considerations

This study used secondary, de-identified data obtained from the Center for Disease Control of Ba Ria Vung Tau Province, including aggregated data on reported dengue cases and meteorological information. Since the research did not involve direct contact with human participants or access to personally identifiable information, it did not require approval from an Institutional Review Board or Ethics Review Committee. However, all data collection and analysis procedures were conducted in accordance with relevant guidelines and regulations for handling epidemiological data to ensure compliance with ethical standards.

## 3. Results

### 3.1. Trends and Fluctuations in Annual Dengue Incidence (2003–2022)

**Figure 2:**
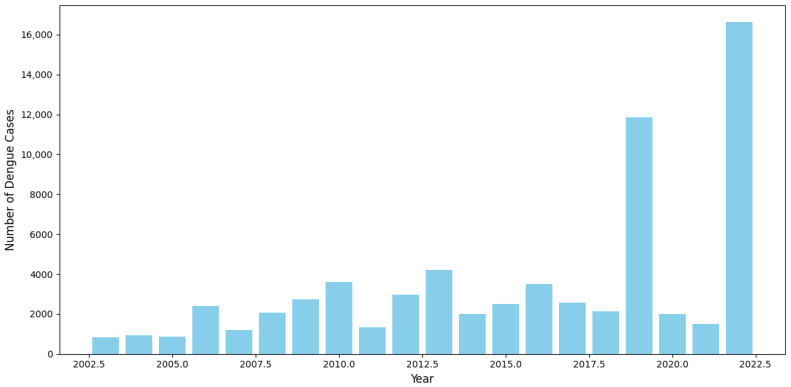
Annual distribution of dengue cases in Ba Ria Vung Tau, Vietnam (2003–2022).

The presented Figure 2 chart highlights the yearly distribution of dengue cases from 2003 to 2022, offering significant insights into the trends and fluctuations of the disease. Over the years, the overall trend indicates a progressive increase in the number of dengue cases, with notable peaks occurring in specific years. In the early period (2003–2010), the number of cases exhibited a gradual increase, reflecting the growing prevalence of the disease. However, between 2010 and 2017, the trend stabilized, with minor fluctuations observed across the years.

The most prominent spike occurred in 2019, when the number of dengue cases surged dramatically, reaching the highest level recorded up to that point. This was followed by an unexpectedly sharp decline in 2020 and 2021. The significant reduction in cases during these years may be attributed to external factors, such as global public health interventions or behavioral changes linked to the COVID-19 pandemic, which potentially mitigated the spread of vector-borne diseases. However, the data for 2022 show a striking resurgence, with the number of cases surpassing even the 2019 peak, suggesting a renewed outbreak with potentially more severe implications.

The dual-axis chart employs both bar and line graphs, allowing for a clear comparison between annual trends and individual fluctuations in case numbers. The consistency between the two visual representations underscores the accuracy of the observed peaks and troughs over time.

In summary, this chart reveals an alarming long-term upward trend in dengue incidence, punctuated by periodic outbreaks, particularly in 2019 and 2022. The data suggest that without effective, sustained public health interventions, the frequency and intensity of dengue outbreaks may continue to rise, posing a significant challenge to global health systems. These findings emphasize the urgent need for reinforced surveillance, prevention, and control measures to curb the further spread of dengue fever.

### 3.2. Descriptive Statistics

**Figure 3:**
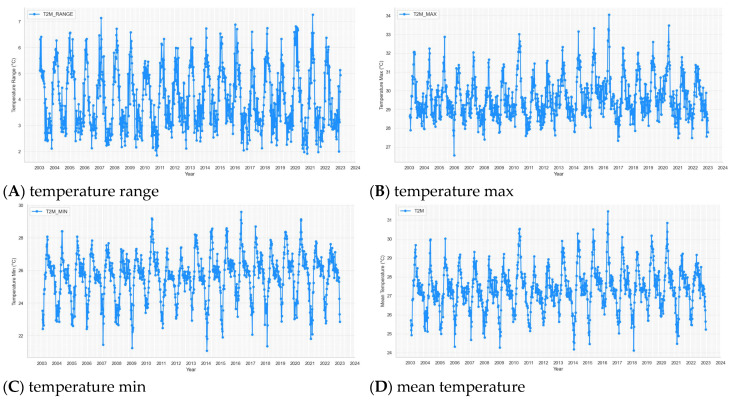

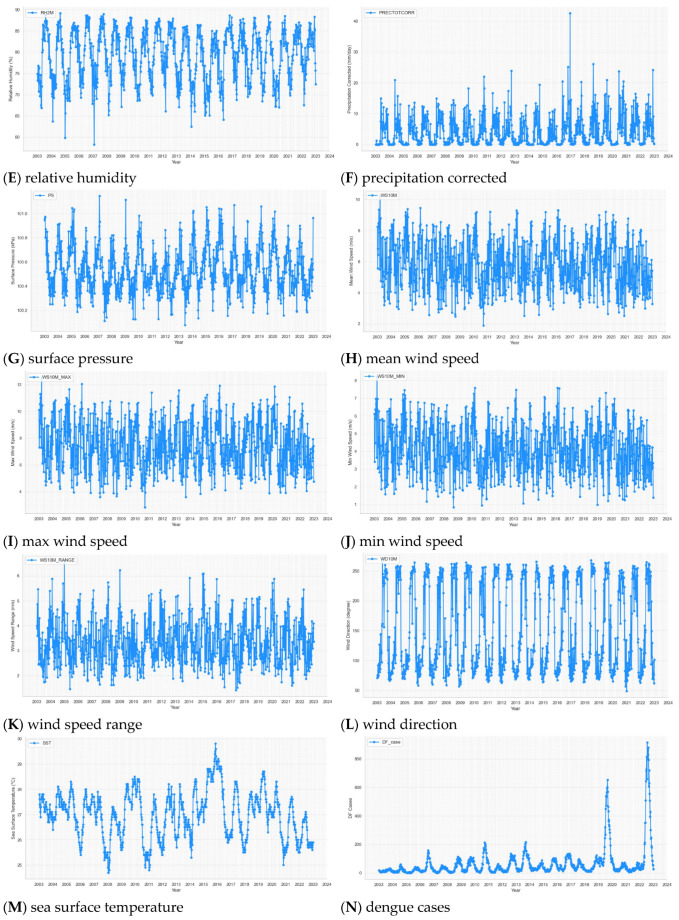
Time series plots of weekly climate variables and dengue cases from week 1—2003 to week 53—2022.

The time series plots of weekly 13 weather variables from 2003 to 2022 of BRVT are shown in Figure 3. Table 2 provides a descriptive summary of the key variables observed in the dataset. The weekly dengue cases (DF_case) show a high level of variability, with an average of 64.94 cases per week and a large standard deviation of 111.99, indicating significant fluctuations in case numbers across different weeks. The minimum number of weekly cases is 0, while the maximum recorded is 913, with 75% of weeks having fewer than 68 cases. This suggests that dengue outbreaks can be sporadic but severe in certain weeks. Temperature-related variables exhibit more stability. The mean maximum temperature (T2M_MAX) is 29.63 °C, with a standard deviation of 1.08 °C, while the minimum temperature (T2M_MIN) averages 25.63 °C, and the average temperature (T2M) stands at 27.38 °C. The temperature range (T2M_RANGE) fluctuates from 1.86 °C to 7.26 °C, indicating relatively mild variability in daily temperature swings across weeks. Humidity levels (RH2M) are generally high, with a mean of 79.28% and a range from 58.29% to 89.13%, reflecting the tropical climate conducive to mosquito breeding. Precipitation (PRECTOTCORR) is highly variable, with a mean of 4.15 mm but a large standard deviation of 4.78 mm, suggesting that rainfall can be inconsistent, with some weeks experiencing no rainfall and others reaching up to 42.53 mm. Wind speed (WS10M) averages 5.76 m/s, with the maximum wind speed (WS10M_MAX) reaching 12.51 m/s and the minimum wind speed (WS10M_MIN) dropping as low as 0.84 m/s. Wind direction (WD10M) has considerable variability, with an average of 154.46 degrees and a range extending from 49.03 to 269.12 degrees. Finally, sea surface temperature (SST) is relatively stable, with a mean of 27 °C and a range from 24.7 °C to 29.8 °C. These environmental factors, temperature, humidity, wind, and precipitation, are critical in understanding the transmission dynamics of dengue, as they directly influence mosquito populations and behavior.

### 3.3. Correlation Analysis

**Figure 4:**
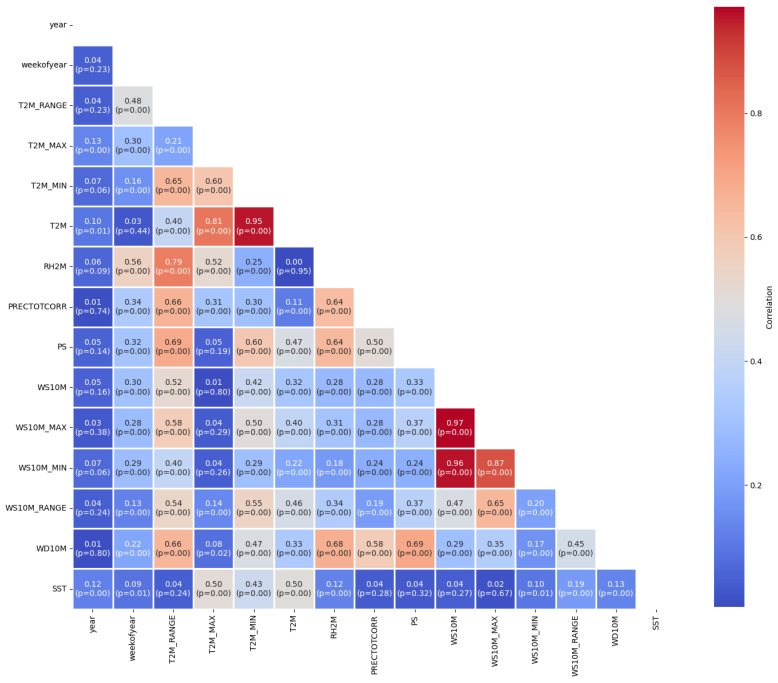
Correlation matrix of climatic variables.

The Pearson correlation analysis in Figure 4 provides insights into the relationships between various climatic variables relevant to the study. A strong positive correlation is observed between WS10M and WS10M_MAX (r = 0.97, *p* < 0.01), indicating that wind speed and its maximum values tend to move together. Similarly, T2M and T2M_MIN exhibit a strong positive correlation (r = 0.95, *p* < 0.01), suggesting that daily mean and minimum temperatures are highly related. Conversely, a moderate negative correlation is seen between T2M_RANGE and T2M_MAX (r = −0.48, *p* < 0.01), implying that greater fluctuations in temperature range are associated with lower maximum temperatures. Additionally, PS shows weaker but notable negative correlations with several temperature-related variables, such as T2M_RANGE (r = −0.32, *p* < 0.01), indicating that higher pressure might be linked with smaller temperature variations. PRECTOTCORR is moderately correlated with T2M_MIN (r = 0.66, *p* < 0.01) and RH2M (r = 0.64, *p* < 0.01), highlighting that temperature, particularly the minimum temperature, and humidity are key drivers of precipitation patterns. This relationship is further supported by a positive correlation between RH2M and T2M_MIN (r = 0.52, *p* < 0.01), confirming the role of temperature in influencing humidity levels.

**Figure 5:**
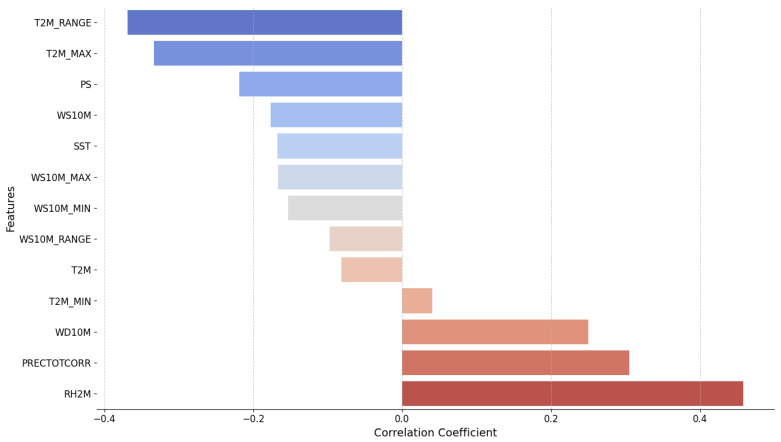
Correlation of climatic variables (features) with total cases. The bar chart illustrates the correlation coefficients of various climatic variables with the total number of cases. The colors represent the direction and strength of the correlation: shades of blue indicate negative correlations, while shades of red indicate positive correlations. Darker colors reflect stronger correlations. This visual distinction helps in understanding how each variable is associated with the total number of cases, with the length of the bars representing the magnitude of the correlation.

The correlation analysis between climatic variables and total cases is presented in Figure 5. The most positively correlated factor with total cases is the RH2M, with a correlation coefficient of approximately 0.4. This suggests that an increase in relative humidity is closely associated with a rise in total cases. Additionally, PRECTOTCORR, WD10M, and T2M_MIN also show positive correlations, albeit weaker. On the other hand, the daily T2M_RANGE has the strongest negative correlation (~−0.35), indicating that an increase in this variable tends to reduce the total cases. Other factors, such as T2M_MAX, PS, and WS10M, also exhibit negative correlations, but with less pronounced effects. Some factors, like SST, WS10M_RANGE, and T2M, have very weak or almost negligible correlations with total cases. Therefore, we will focus on the following variables as they provide the highest correlations, including RH2M, PRECTOTCORR, WD10M, T2M_RANGE, T2M_MAX, and PS, and further examine the correlation of their lagged versions with total cases.

The analysis of the climatic factors reveals distinct relationships between specific variables and the total cases (Figure S1). Temperature-related variables demonstrate a notable negative correlation with case counts. Specifically, as the temperature range increases, the total number of cases decreases, indicating that larger variations in daily temperature may contribute to a reduction in cases. Similarly, an increase in maximum temperature is associated with a slight decline in case numbers, although the effect is less pronounced. Relative humidity, on the other hand, shows a clear positive correlation with the total number of cases. Higher humidity levels are consistently linked with an increase in cases, suggesting that humidity could play a significant role in disease transmission or exacerbation. Precipitation does not display a consistent trend; however, there is a marked increase in cases at certain points, followed by fluctuations, which implies a possible non-linear or threshold-dependent relationship. Surface pressure appears to have a negligible effect, as total cases remain relatively constant across varying pressure levels. Finally, wind direction exhibits considerable variability, with no discernible trend, indicating that its influence on case counts may be highly context-specific or modulated by other factors.

Figure S2 shows that the variables with the highest correlation for total cases are T2M_RANGE_shift_14, T2M_MAX_shift_20, RH2M_shift_6, PRECTOTCORR_shift_10, PS_shift_16, and WD10M_shift_14.

### 3.4. Negative Binomial Regression (NBR)

Negative Binomial Regression (NBR) was the chosen method in this study to predict the number of dengue cases (total_cases). The target variable is a count variable, and the dataset exhibited overdispersion (variance much larger than the mean), which makes NBR a more appropriate model than Poisson regression.

Distribution analysis of the target variable showed that the mean number of dengue cases was 43.73, while the variance was 1692.84, confirming that the data had high variability (Figure S3). The histogram of the target variable showed a right-skewed distribution, with most values concentrated on the left and tapering off toward the right. The KDE curve (Figure S3) also reflected this dispersion, which aligns with the negative binomial distribution, where the probability of occurrence decreases as the number of cases increases.

The first negative binomial regression model was built with six initial variables. The results indicated that the model had an optimal alpha of approximately 0.034, with a Mean Absolute Error (MAE) of 26.035 on the test set and 26.179 on the training set (Table 3 and Figure 12A). The small difference between these MAE values suggests that the model does not overfit and generalizes well.

In the second model, additional variables were included, such as the time variable (month), which significantly improved the model’s performance. The optimal alpha decreased to 0.022, and the MAE values for both the training and test sets dropped to 25.916 and 25.556, respectively. This indicates an improvement in predictive accuracy (Figure 12B).

The third model introduced time-shifted variables, which further improved prediction accuracy, reducing the test set MAE to 24.845. Time-shifted variables helped the model better capture temporal trends and fluctuations in the data, improving its overall performance (Figure 12C).

Finally, the fourth model, which incorporated time-shifted variables with lag, produced the best prediction results, with an alpha = 1.95. The MAE for the test set decreased to 21.409, significantly lower than the previous models. This improvement indicates that using lagged variables allowed the model to capture more complex fluctuations in the data, resulting in more accurate predictions (Figure 12D).

**Figure 6:**
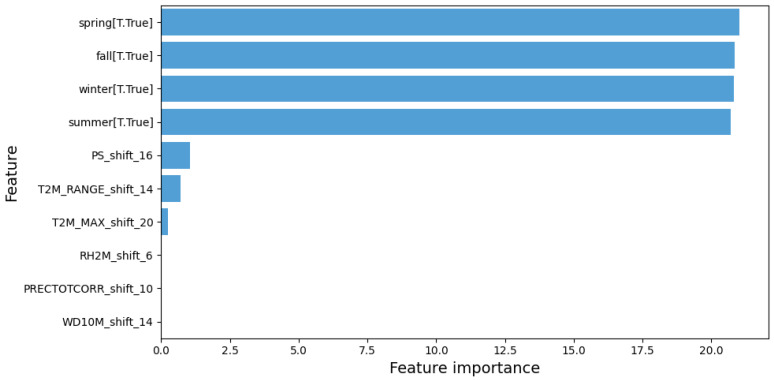
Relative importance of features in predicting dengue cases.

In summary, the negative binomial regression model with time-shifted and lagged variables demonstrated superior performance, offering more accurate predictions than the previous models. Climate variables such as T2M_RANGE_shift_14 and PS_shift_16 were identified as crucial factors in predicting dengue cases, along with seasonal factors (Figure 6).

### 3.5. SARIMAX Model

**Figure 7:**
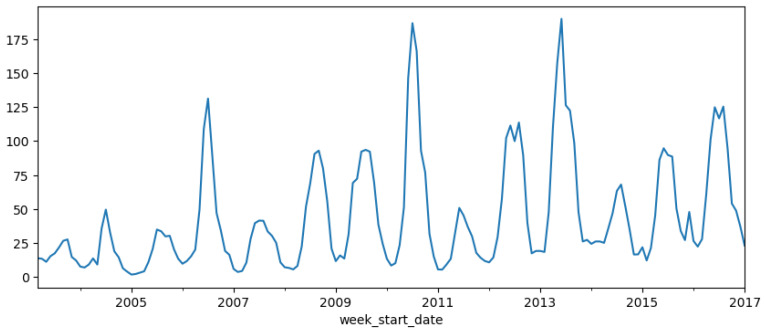
Time series of monthly dengue cases from the training set.

To address seasonality and autocorrelation in the dataset, the SARIMAX model was selected to predict dengue cases. The data were resampled from weekly to monthly, as hyperparameter tuning using pm.auto_arima was not effective with weekly data (Figure 7).

**Figure 8:**
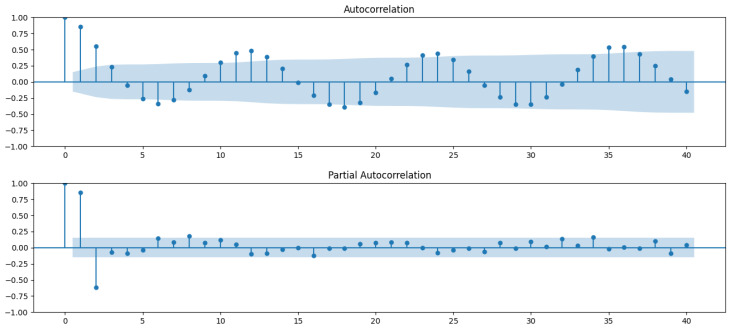
ACF (**top**) and PACF (**bottom**) plots. The blue shaded area in both plots represents the 95% confidence interval. Autocorrelation (or partial autocorrelation) values falling within the shaded area are not statistically significant, indicating that they may occur by chance. Values outside this region suggest statistically significant correlations at those lags.

The analysis began with Autocorrelation Function (ACF) and Partial Autocorrelation Function (PACF) plots to assess the degree of autocorrelation within the time series. These plots helped determine the order of the AutoRegressive (AR) and Moving Average (MA) terms. The PACF suggested that the AR order (*p*) could be 1 or 2, and the ACF indicated the MA order (q) could be 1, 2, or 3 (Figure 8).

Next, seasonal decomposition was applied to examine the trend and seasonality of the data, which clearly showed a seasonal pattern with no strong trend (Figure S4). A stationarity test confirmed that the time series was stationary, which allowed d and D to be set to 0 in the SARIMAX model.

#### 3.5.1. SARIMAX #1 Baseline Model

**Figure 9:**
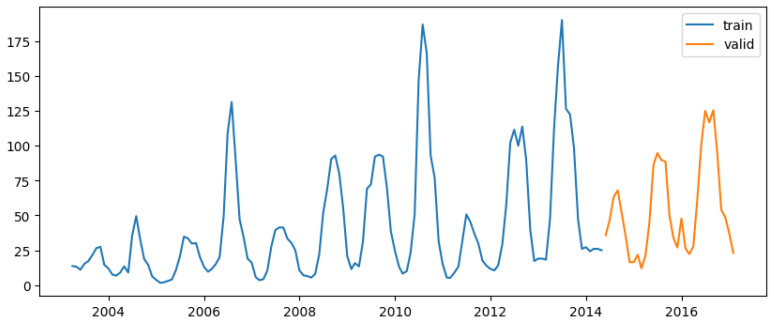
Train–test data split for all models in this study.

Additionally, 80% of the data allocated for training and 20% for testing (Figure 9) are applied to all models in this study. The first SARIMAX model used only total_cases as the predictor variable. The auto_arima() function identified the best-fitting model as ARIMA(2 0 0)(1 0 1) [12], which captures both autoregressive and seasonal moving average components (Table S1).

The SARIMAX model fit the data well, with statistically significant AR and MA parameters, capturing the autocorrelation and seasonality of the dataset. However, diagnostic tests revealed that while the model fit was good (no significant autocorrelation left in the residuals), the residuals showed positive skewness and high kurtosis (Table S1, Figure S5). This suggests that some outliers or non-normal distribution of residuals may be present.

The MAE and RMSE scores for the training and test sets highlight a significant overfitting issue: MAE_train = 10.54 vs. MAE_test = 20.31, and RMSE_train = 16.10 vs. RMSE_test = 27.19 (Figure 12E). The model performed well on the training data but struggled with the test data, indicating that the baseline SARIMAX model is overfitted.

#### 3.5.2. SARIMAX #2 Full Multivariate Model

A second SARIMAX model was developed using lagged exogenous variables such as T2M_RANGE_shift_14, T2M_MAX_shift_20, RH2M_shift_6, and others as predictors (Figure 9). The best model identified was ARIMA(2 0 0)(1 0 0) [12].

However, the model did not converge, suggesting that either the parameter orders or the initial parameter values were not optimal. The statistical results (Table S2) showed that most independent variables were not statistically significant, with the exception of a few AR parameters. Despite the improved model, it still exhibited issues such as heteroskedasticity and residuals not following a normal distribution (Figure S6).

The MAE and RMSE scores for SARIMAX #2, MAE_train = 11.37 and MAE_test = 17.02, showed a slight improvement compared to the baseline model. However, the model still failed to predict the test data accurately (Figure 12F).

### 3.6. XGBoost Regression

#### 3.6.1. Model #1

For the first XGBoost regression model, we used the original variables: total_cases, month, T2M_RANGE, T2M_MAX, RH2M, PRECTOTCORR, PS, and WD10M. The dataset was split into 80% training and 20% testing sets, with a 5-fold cross-validation performed (Figure 12G). Hyperparameter tuning was conducted using GridSearchCV, which was optimized for neg_mean_squared_error during cross-validation. The best parameters obtained were as follows: colsample_bytree: 0.5, gamma: 1, learning_rate: 0.2, max_depth: 8, min_child_weight: 3, objective: reg, subsample: 1.

The Mean Absolute Error (MAE) for the best model was −1596.43, and the best score showed that the model had overfitting issues, with a much lower error on the training set than the test set. Specifically, the training MAE was 1.07, while the test MAE was 21.77. Similarly, the Root Mean Squared Error (RMSE) was 1.46 for training and 29.73 for testing, further highlighting overfitting (Figure 12G).

These results suggest that the model fits the training data well but fails to generalize to new data, which is likely due to overfitting caused by a high max_depth and min_child_weight, which made the model overly complex.

#### 3.6.2. Model #2

In the second model, we introduced lagged variables such as T2M_RANGE_shift_14, T2M_MAX_shift_20, RH2M_shift_6, PRECTOTCORR_shift_10, and others (Figure 12H). The best parameters for this model were as follows: colsample_bytree: 0.75, gamma: 1, learning_rate: 0.2, max_depth: 4, min_child_weight: 1, objective: reg, subsample: 0.75.

The MAE for the best model was −1355.61, and while the performance improved slightly, the model still exhibited overfitting. The training MAE was 6.63, while the test MAE increased to 24.45. The RMSE was 13.04 for training and 30.97 for testing (Figure 12H).

After refitting the model using the entire dataset, predictions showed two additional dengue peaks in early 2018 and 2022 (Figure 12I and Figure S7).

**Figure 10:**
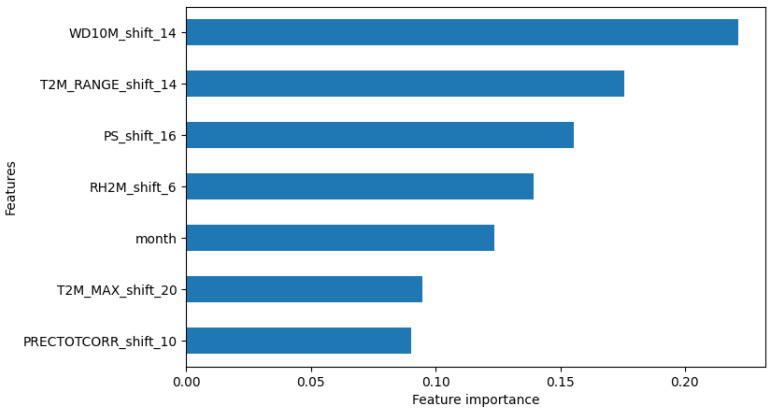
Relative feature importance in predicting dengue cases for XGB Regression Model #2.

In terms of feature importance, WD10M_shift_14 (14-week lagged wind direction) and T2M_RANGE_shift_14 (14-week lagged temperature range) were identified as the most important variables for predicting dengue cases (Figure 10).

### 3.7. LSTM Neural Network

Regarding the LSTM model, the input data were structured as a 3D tensor with shape (batch_size, timesteps, input_dim). The data preprocessing involved scaling and converting both the training and testing sets using a scaler before applying predictions (Figure 9).

#### 3.7.1. LSTM Model #1

**Figure 11:**
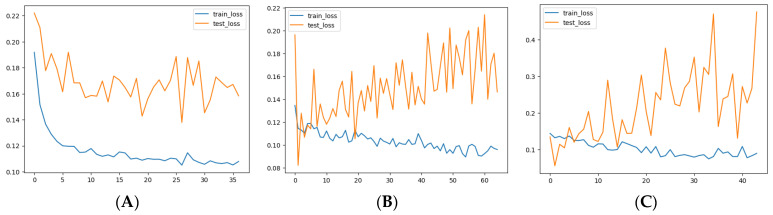
Loss convergence on training and test sets for LSTM: (**A**) Model #1; (**B**) Model #2; (**C**) Model #3.

**Figure 12:**
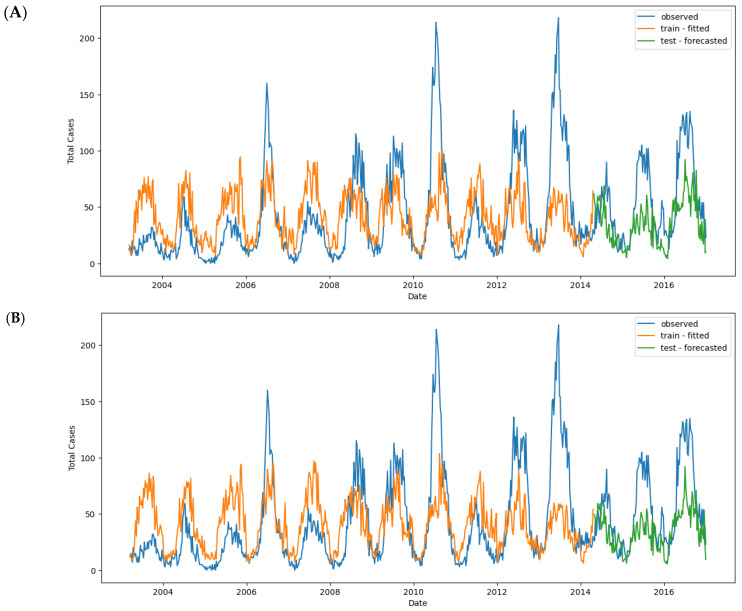

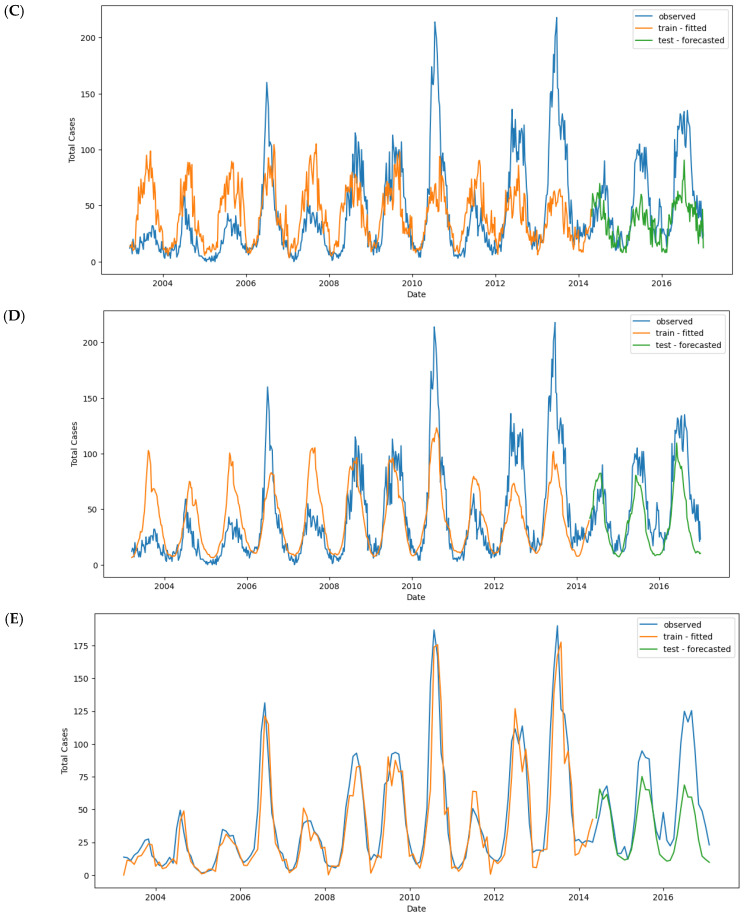

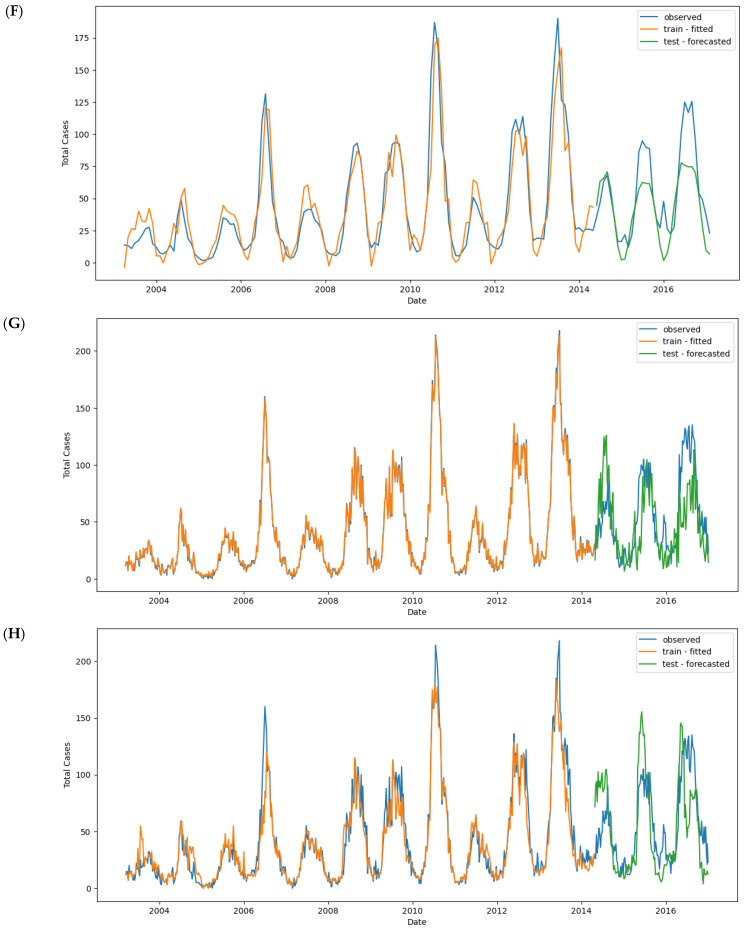

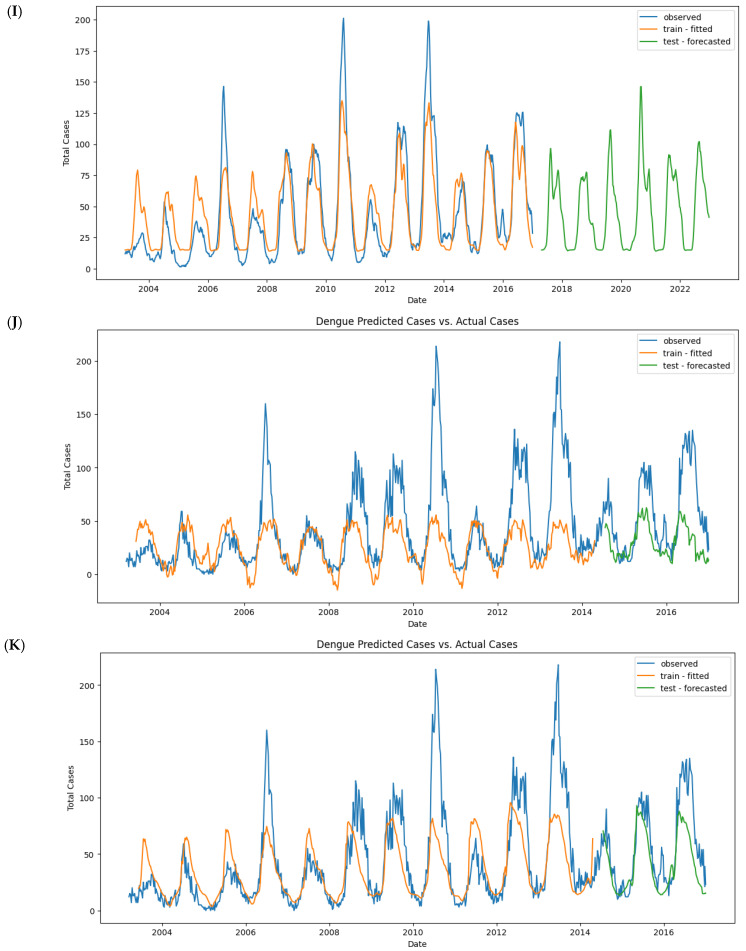

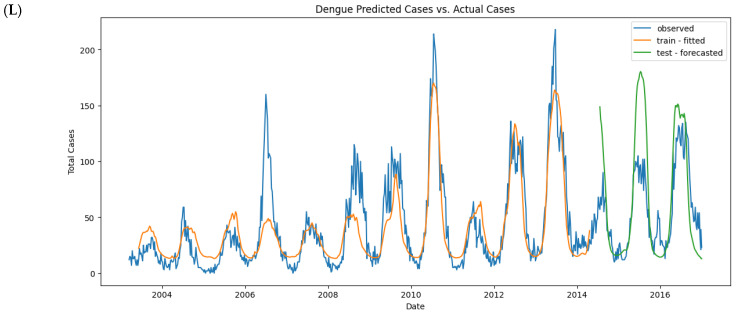
Observed and predicted dengue cases in training (week 1 of 2003 to week 17 of 2017) and test data (week 18 of 2017 to week 53 of 2022) of different models: (**A**) NBR #1; (**B**) NBR #2; (**C**) NBR #3; (**D**) NBR #4; (**E**) SARIMAX #1; (**F**) SARIMAX #2; (**G**) XGBoost #1; (**H**) XGBoost #2; (**I**) XGBoost #2 (refit); (**J**) LSTM #1; (**K**) LSTM #2; (**L**) LSTM #3.

A simple LSTM model with a single hidden layer of LSTM units and one output layer was implemented. After 35 epochs, the model showed convergence in both the training and testing sets, as visualized in Figure 11A. However, despite the convergence, the model’s performance metrics indicate room for improvement. The MAE was 23.73 for the training set and 28.86 for the test set. The RMSE was 38.03 for the training set and 38.65 for the test set (Figure 12J).

While the model captured the overall seasonality of the data, it failed to detect individual peaks and outbreaks in both the training and test sets. This suggests that a more complex architecture is required to capture these finer details.

#### 3.7.2. LSTM Model #2

A deeper LSTM model with more layers and neurons was trained to improve performance. While the model converged well on the training data (Figure 11B), it struggled to generalize to the test set, as evidenced by fluctuations in the test loss. The MAE and RMSE slightly improved but remained unsatisfactory, with MAE_train at 18.70 and MAE_test at 18.14. The RMSE was 29.92 for training and 24.37 for testing (Figure 12K).

This model showed some improvement, but it still could not capture individual peaks or outbreaks, which suggests the need for additional tuning or alternative input features.

#### 3.7.3. LSTM Model #3

In this iteration, lagged variables were introduced into the model, which included total_cases, month, T2M_RANGE_shift_14, T2M_MAX_shift_20, RH2M_shift_6, PRECTOTCORR_shift_10, PS_shift_16, and WD10M_shift_14. Despite adding these features, the model encountered difficulty generalizing on the test set, as shown in Figure 11C. The MAE on the training set was 13.89, while the test MAE increased to 24.86. Similarly, the RMSE was 20.54 for training and 34.92 for testing (Figure 12L).

Despite using a more complex LSTM model and incorporating lagged variables, the model still struggled to predict the large values in the test set accurately. While the model improved over the previous versions, it still failed to detect individual peaks or outbreaks in the data.

### 3.8. Assessment of Predictive Performance

This section evaluates the predictive performance of four models—NBR, SARIMAX, XGBoost, and LSTM Neural Network—in predicting dengue cases. Key performance metrics include Mean Absolute Error (MAE) and Root Mean Squared Error (RMSE), which were computed for both the training and testing datasets. Table 4 below summarizes the performance of each model.

Each of the four models showed distinct advantages and limitations in predicting dengue cases. NBR demonstrated steady improvement across all iterations, with Model #4 providing the most balanced performance, showing improvements in both predictive accuracy and generalization. SARIMAX performed well in capturing seasonality but suffered from overfitting, particularly in Model #1, while Model #2 reduced this issue but still required further refinement to enhance test performance. XGBoost demonstrated excellent performance on training data but failed to generalize well to unseen data due to overfitting, despite slight improvements with lagged variables in Model #2. LSTM showed potential in capturing temporal dependencies and seasonality, especially in Model #2, but consistently struggled with generalizing to test data and accurately predicting outbreaks or peaks. In summary, NBR Model #4 emerged as the most reliable and well-rounded model for dengue case prediction, whereas SARIMAX and XGBoost models could benefit from further optimization. LSTM, despite its promise for long-term temporal predictions, requires additional refinement to effectively capture short-term peaks and outbreak patterns.

## 4. Discussion

The results of this study align with previous research, reinforcing the critical role of climate variables such as temperature, humidity, and precipitation in driving dengue transmission. Notably, RH2M, PRECTOTCORR, and T2M_RANGE, T2M_MAX were identified as key factors affecting dengue incidence in BRVT. These findings are consistent with studies conducted in other tropical regions and have highlighted the influence of climate conditions on mosquito breeding and virus transmission.

Several studies have confirmed that climatic factors such as temperature and humidity play a pivotal role in shaping the dynamics of dengue outbreaks. For instance, research in Southern China demonstrated that higher temperatures and humidity are directly correlated with increases in mosquito populations, which in turn escalates dengue transmission [1]. Similarly, a Bayesian analysis in Queensland, Australia, found that increases in rainfall and higher temperatures significantly raised the likelihood of dengue outbreaks [3]. Our study corroborates these findings, with RH2M and PRECTOTCORR both showing strong positive correlations with dengue cases in BRVT, suggesting that humid and wet conditions provide favorable environments for Aedes aegypti mosquitoes, the primary vector for dengue.

In comparison to models used in other regions, such as Brazil and Mexico, where dynamic ensemble learning and multi-stage machine learning models have been effectively utilized to predict dengue outbreaks [5], our study introduces a novel approach by integrating both meteorological data and time-lagged variables. This approach allows for the modeling of delayed effects of climatic conditions on dengue incidence, which is particularly crucial given the mosquito lifecycle and the virus incubation period.

Among the models evaluated, the Negative Binomial Regression (NBR) model performed the best, particularly when incorporating lagged climate variables, achieving the lowest mean absolute error (MAE) on both the training and test sets. The effectiveness of NBR in handling overdispersed count data aligns with its successful application in similar studies focused on vector-borne diseases. For instance, a study in Mexico employed a multi-stage machine learning approach to predict dengue, with lagged climate data significantly improving model performance [6].

Our study’s use of lagged temperature and humidity variables (e.g., T2M_MAX_shift_20, RH2M_shift_6) reflects a common strategy in dengue forecasting, as demonstrated in research conducted in central Vietnam and Southern China, where lagged climatic variables proved crucial in capturing the delayed impacts of weather on dengue transmission [8]. The inclusion of lagged variables further enhanced the generalization capabilities of our models, reducing overfitting issues observed in other models such as SARIMAX and LSTM.

This is consistent with findings from other studies that applied SARIMA models to time-series data, where the inclusion of external variables often improves the model’s predictive accuracy, as observed in the study of climate-based dengue forecasting in Vietnam [2]. However, the introduction of exogenous variables in our SARIMAX model only provided marginal improvements, indicating the need for further tuning of seasonal components and better feature selection.

On the other hand, the LSTM model, while promising for long-term forecasting, struggled to capture outbreak peaks, which are crucial for timely public health interventions. Similar challenges were observed in other studies applying LSTM models for forecasting dengue, where capturing outbreak peaks remains challenging [10].

The successful application of machine learning techniques such as NBR and XGBoost in this study demonstrates the potential of these models to provide accurate, climate-based early warning systems for dengue outbreaks. Accurate prediction models enable health authorities to implement timely control measures, such as mosquito population reduction and public awareness campaigns, before outbreaks occur. The inclusion of climate variables such as temperature, humidity, and precipitation provides a more nuanced understanding of dengue transmission, aligning with global efforts to integrate meteorological data into vector-borne disease forecasting systems [2].

Consideration of Serotype and Genotype Dynamics: In this study, the predictive models primarily relied on climatic factors, such as temperature, humidity, precipitation, and wind speed, along with lagged variables to account for the delayed effects of weather conditions on dengue transmission. However, the analysis did not incorporate data on dengue virus serotypes and genotypes, which are important factors that can significantly influence the dynamics and severity of outbreaks. The exclusion of these virological factors represents a limitation of the study, as different serotypes can lead to varied immune responses and disease severity, thereby impacting outbreak patterns.

Availability of National Serotype Data in Vietnam: In Vietnam, national data on circulating dengue serotypes is available and could be used to improve the predictive accuracy of the models. The distribution of serotypes changes across regions and over time, influencing the transmission dynamics of the disease. Incorporating serotype data as a predictor would allow the models to account for changes in population immunity and the associated risks due to shifts in serotype dominance, thus providing a more comprehensive approach to forecasting dengue outbreaks.

Recommendations for Future Model Enhancements: To address this limitation, future research should integrate virological data, such as serotype and genotype information, alongside climatic variables. This integration would enable the models to capture not only the environmental drivers of dengue transmission but also the immunological and virological factors that affect outbreak risks. Including these factors could potentially improve the accuracy and responsiveness of dengue early warning systems, particularly in regions where serotype shifts have been observed.

Consideration of Asymptomatic and Underreported Dengue Cases: In this study, the predictive models were based on reported symptomatic dengue cases obtained from the Center for Disease Control of Ba Ria Vung Tau Province. However, this approach does not account for asymptomatic or mildly symptomatic cases that do not seek medical attention, which may lead to an underestimation of the true burden of dengue. It is well-recognized that some dengue virus serotypes are more likely to cause mild or asymptomatic infections, and these cases can go undetected in routine surveillance data. The reliance on reported symptomatic cases represents a limitation of our study, as it does not capture the full extent of dengue transmission in the population. To address this limitation, future research should consider using seroprevalence studies or community-based surveys to estimate the proportion of asymptomatic or unreported cases. Incorporating these estimates into the forecasting models could provide a more accurate representation of the dengue epidemic, particularly during large outbreaks when underreporting may be more pronounced. This approach would allow the models to better account for the hidden burden of dengue and improve the reliability of early warning systems for public health responses.

However, there are limitations to our study. First, the reliance on climate data from NASA/POWER CERES/MERRA2 may introduce uncertainties, particularly with imputation techniques used for missing data. This issue is common in studies using remote sensing data, as gaps in data often require sophisticated imputation methods, which can affect model accuracy [10]. Moreover, while the models were able to capture broad trends in dengue incidence, they were less successful in predicting exact outbreak peaks, particularly in years like 2019 and 2022, where dengue cases spiked unexpectedly. This indicates that additional variables, such as socio-environmental factors and mosquito population dynamics, should be integrated into future models to improve prediction accuracy.

Limitations in Differentiating Arboviral Infections: While laboratory confirmation helps ensure the accuracy of reported dengue cases used in the study, there are still limitations in distinguishing co-infections or misdiagnoses in regions where multiple arboviruses co-circulate. The similarity in clinical presentation between dengue, Chikungunya, and Zika can lead to diagnostic challenges, especially in cases where laboratory resources are limited or only serological tests are performed. To improve future research, expanded diagnostic protocols, including broader RT-PCR testing for multiple arboviruses, could enhance the differentiation between these infections and provide a more accurate picture of the true incidence of each disease.

Future research should focus on integrating additional environmental and socio-demographic factors, such as population density, land use, and mosquito abundance, to further enhance the predictive power of dengue forecasting models. Ensemble approaches, which combine the strengths of multiple models, could also be explored to improve accuracy. Moreover, the application of more sophisticated deep learning architectures, such as hybrid LSTM models, could help overcome challenges in detecting short-term dengue outbreaks, as seen in recent research on vector-borne diseases [10].

## 5. Conclusions

This study has demonstrated the effectiveness of climate-based machine learning models in forecasting dengue outbreaks in BRVT, Vietnam. By leveraging advanced models such as NBR, SARIMAX, XGBoost, and LSTM, we were able to capture key climatic factors—such as temperature, humidity, wind speed, and precipitation—that significantly impact dengue transmission. Among the models tested, the NBR model incorporating lagged climate variables proved to be the most accurate and generalizable in predicting weekly dengue cases, highlighting the value of considering delayed climate effects in disease forecasting.

While this research adds to the growing body of literature supporting the integration of meteorological data into public health models, there remain challenges, particularly in capturing short-term dengue outbreaks. Models like SARIMAX and LSTM exhibited overfitting and struggled with predicting peak outbreaks. This suggests the need for further refinement of these models, possibly through the inclusion of additional socio-environmental factors and ensemble modeling approaches.

## Figures and Tables

**Table 1:** Climate parameters used in the models of this study.

No.	Parameter	Symbol	Unit
1	Temperature at 2 M Range	T2M_RANGE	°C
2	Temperature at 2 M Maximum	T2M_MAX	°C
3	Temperature at 2 M Minimum	T2M_MIN	°C
4	Temperature at 2 M	T2M	°C
5	Relative Humidity at 2 M	RH2M	%
6	Precipitation Corrected	PRECTOTCORR	mm/day
7	Surface Pressure	PS	kPa
8	Wind Speed at 10 M	WS10M	m/s
9	Wind Speed at 10 M Maximum	WS10M_MAX	m/s
10	Wind Speed at 10 M Minimum	WS10M_MIN	m/s
11	Wind Speed at 10 M Range	WS10M_RANGE	m/s
12	Wind Direction at 10 M	WD10M	Degrees
13	Sea Surface Temperature	SST	°C

**Table 2:** Descriptive statistics of weekly weather variables and dengue cases from 2003 to 2022 in Ba Ria Vung Tau, Vietnam.

Variables	Count	Mean	Std	Min	25%	50%	75%	Max
DF_case	1044	64.943	111.994	0	15	32	68	913
T2M_RANGE	1044	3.998	1.166	1.86	3.057	3.769	4.926	7.263
T2M_MAX	1044	29.63	1.082	26.57	28.852	29.427	30.242	34.044
T2M_MIN	1044	25.633	1.404	21.071	24.863	25.826	26.527	29.596
T2M	1044	27.376	1.131	24.126	26.768	27.34	28.012	31.453
RH2M	1044	79.275	5.937	58.286	74.646	80.393	84.312	89.134
PRECTOTCORR	1044	4.145	4.783	0	0.197	2.457	6.729	42.527
PS	1044	100.538	0.184	100.08	100.407	100.509	100.661	101.144
WS10M	1044	5.764	1.538	1.897	4.631	5.656	6.936	10.234
WS10M_MAX	1044	7.406	1.772	2.851	6.064	7.304	8.776	12.514
WS10M_MIN	1044	4.093	1.367	0.844	3.106	4.004	5.099	8.21
WS10M_RANGE	1044	3.313	0.88	1.42	2.657	3.2	3.896	6.61
WD10M	1044	154.463	72.843	49.03	86.743	123.339	239.295	269.124
SST	1044	26.999	0.934	24.7	26.3	27.1	27.6	29.8

**Table 3:** Generalized linear model regression results for Model 1.

	Coef	Std Err	z	*p* > |z|	[0.025	0.975]
Intercept	20.2737	10.862	1.866	0.062	−1.016	41.563
T2M_RANGE	−0.1264	0.017	−7.242	0	−0.161	−0.092
T2M_MAX	−0.2771	0.016	−16.913	0	−0.309	−0.245
RH2M	0.0563	0.005	12.386	0	0.047	0.065
PRECTOTCORR	−0.0141	0.003	−4.037	0	−0.021	−0.007
PS	−0.1235	0.104	−1.185	0.236	−0.328	0.081
WD10M	−0.0004	0	−1.615	0.106	−0.001	8 × 10^−5^

**Table 4:** Summary of predictive performance for NBR, SARIMAX, XGBoost, and LSTM models.

Model	MAE (Train)	MAE (Test)	RMSE (Train)	RMSE (Test)	Key Observations
NBR #1	26.321	25.822	37.380	33.413	Reasonable generalization but struggles with larger fluctuations.
NBR #2	25.916	25.556	37.397	33.502	Slight improvement; still lacks peak detection.
NBR #3	25.426	24.846	37.101	32.364	Better generalization, but key outbreak peaks remain undetected.
NBR #4	20.748	21.409	29.755	26.427	Significant improvement in predictive accuracy and generalization.
SARIMAX #1	10.539	20.307	16.101	27.190	Strong overfitting; poor generalization to test data.
SARIMAX #2	11.367	17.017	15.786	22.635	Reduced overfitting; still requires fine-tuning for better test performance.
XGBoost #1	1.074	21.767	1.461	29.732	Severe overfitting; excellent train performance but poor test generalization.
XGBoost #2	6.631	24.450	13.035	30.973	Minor improvement with lagged variables; overfitting persists.
LSTM #1	23.731	28.856	38.031	38.650	Captures seasonality but fails to detect individual peaks or outbreaks.
LSTM #2	18.704	18.143	29.922	24.368	Deeper model improves fit, but test performance remains inconsistent.
LSTM #3	13.890	24.859	20.544	34.920	Improved training results, but test performance is weak, especially with peaks.

## Data Availability

The data presented in this study are available on request from the corresponding author. The data are not publicly available due to privacy and ethical restrictions, as they contain sensitive health information related to individuals affected by the disease. Anonymized data may be made available upon reasonable request and with permission from the Center for Disease Control of Ba Ria Vung Tau Province, Vietnam.

## Supplementary Materials

The following supporting information can be downloaded at: https://www.mdpi.com/article/10.3390/tropicalmed9100250/s1, Figure S1: Impact of climatic factors on total cases; Figure S2: Correlations of selected climate variables at cumulative mean values of lags from 2 to 20 weeks (approximately 3–4 months), using a minimum period of 10 for a lag of 20 so as not to lose all data of the first 20 weeks. (A) T2M_RANGE and T2M_MAX, (B) RH2M, (C) PRECTOTCORR, (D) PS, (E) WD10M; Figure S3: Distribution of the target variable (total cases); Figure S4: Seasonal and trend decomposition of the dengue case time series; Figure S5: SARIMAX #1 Residual diagnostics; Figure S6. SARIMAX #2 Residual diagnostics; Figure S7: Zoomed-in forecast showing additional dengue peaks for XGB Regression Model #2; Table S1: Parameter estimation and statistical diagnostics of SARIMAX #1; Table S2: Parameter estimation and statistical diagnostics of SARIMAX #2.
