# Correction: Tuan, D.A. Leveraging Climate Data for Dengue Forecasting in Ba Ria Vung Tau Province, Vietnam: An Advanced Machine Learning Approach. *Trop. Med. Infect. Dis*. 2024, *9*, 250

**DOI:** 10.3390/tropicalmed10050144

**Published:** 2025-05-21

**Authors:** Dang Anh Tuan

**Affiliations:** Faculty of Public Health, University of Medicine and Pharmacy at Ho Chi Minh City, Ho Chi Minh City 70000, Vietnam; tuanda222@gmail.com

Following the initial publication [1], one of the listed co-authors, Tran Ngoc Dang, requested the removal of his name from the authorship. The request was based on his reassessment of his level of contribution, as he clarified that he did not participate directly in the manuscript writing or the editorial process of this paper. After internal discussion and mutual understanding, the author agreed with this request in alignment with the authorship criteria outlined by the journal. The Author Contributions section is deleted.

A correction has been made to the Acknowledgments statement. The corrected Acknowledgments statement appears here:

**Acknowledgments:** The author would like to express his sincere gratitude to all members of the research team of the University of Medicine and Pharmacy at Ho Chi Minh City, Vietnam, for their invaluable contributions to this study. The author extends his deepest appreciation to the Pasteur Institute in Ho Chi Minh City, Vietnam, for providing critical data and insights that were instrumental in developing the dengue risk prediction framework. Additionally, the author acknowledges the support and cooperation of public health officers, community leaders, and healthcare professionals in Ba Ria Vung Tau Province, Vietnam, whose efforts in data collection and community engagement greatly enriched the quality and relevance of this research. This study would not have been possible without the collaborative spirit and dedication of all involved parties.

The author states that the scientific conclusions are unaffected. This correction was approved by the Academic Editor. The original publication has also been updated.
