# Supplementary material for: Leveraging Climate Data for Dengue Forecasting in Ba Ria Vung Tau Province, Vietnam: An Advanced Machine Learning Approach
Source: Trop Med Infect Dis. 2024 Oct 21;9(10):250. doi: 10.3390/tropicalmed9100250 (PMC11511084; doi:10.3390/tropicalmed9100250)
Supplement: Supplementary file 1 [file tropicalmed-09-00250-s001.zip › tropicalmed-3245467-supplementary.pdf]

# Leveraging Climate Data for Dengue Forecasting in Ba Ria Vung Tau Province, Vietnam: An Advanced Machine Learning Approach

## Supplementary file:

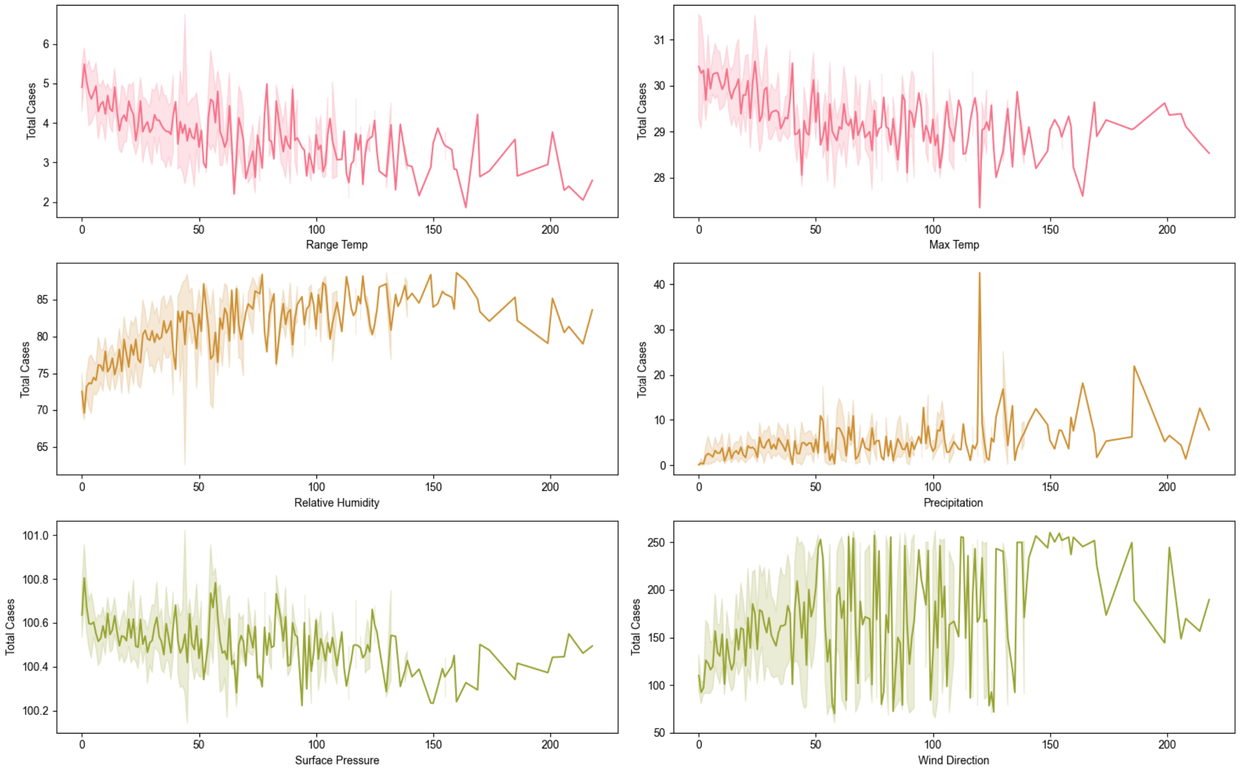


**Figure S1.** Impact of climatic factors on total cases.

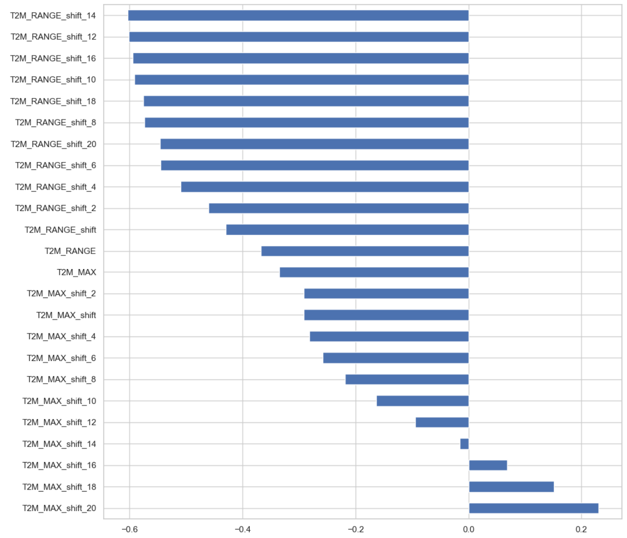


(A)

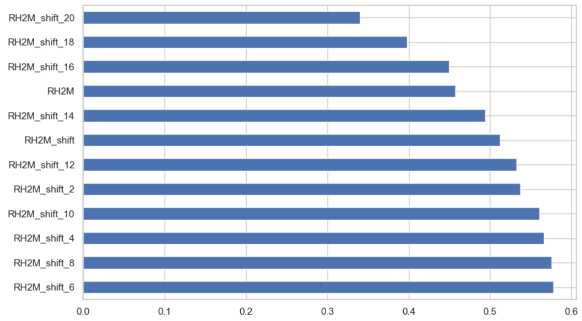


(B)

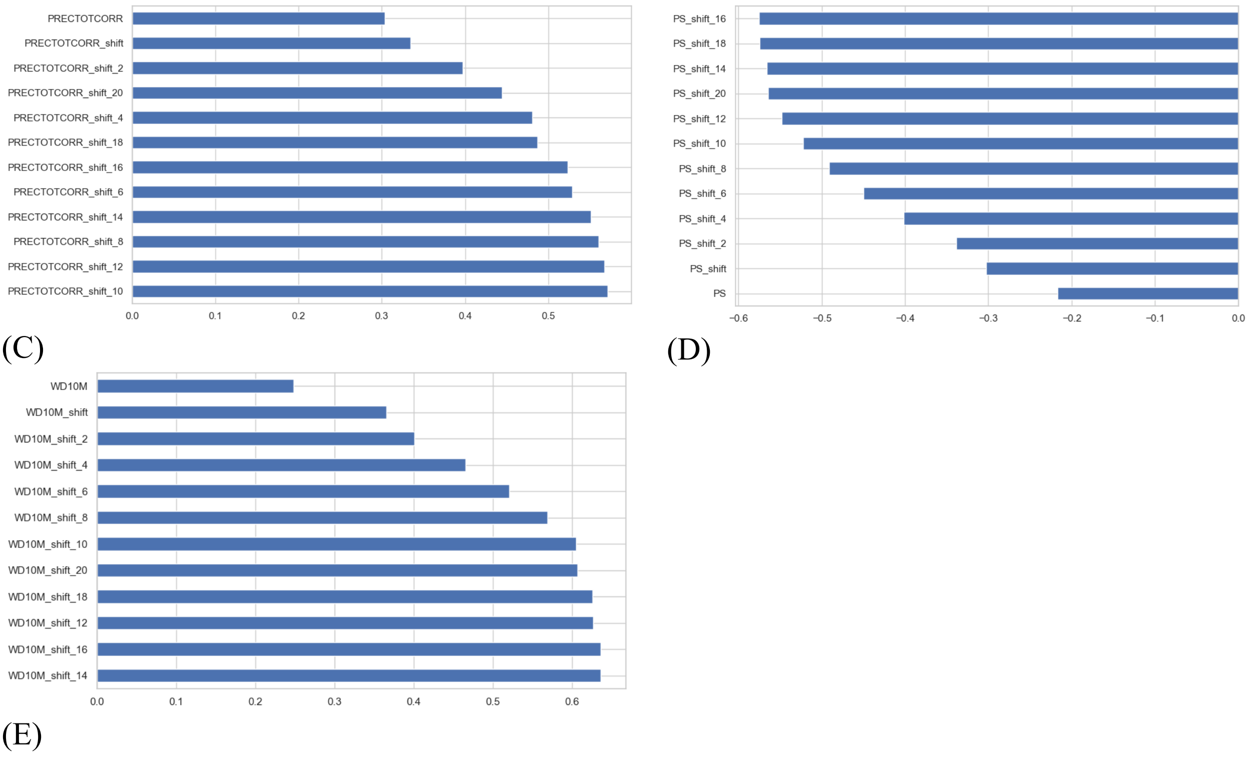


**Figure S2.** Correlations of selected climate variables at cumulative mean values of lags from 2 to 20 weeks (approximately 3 - 4 months), using a minimum period of 10 for a lag of 20 so as not to lose all data of the first 20 weeks. (A) T2M_RANGE and T2M_MAX, (B) RH2M, (C) PRECTOTCORR, (D) PS, (E) WD10M.

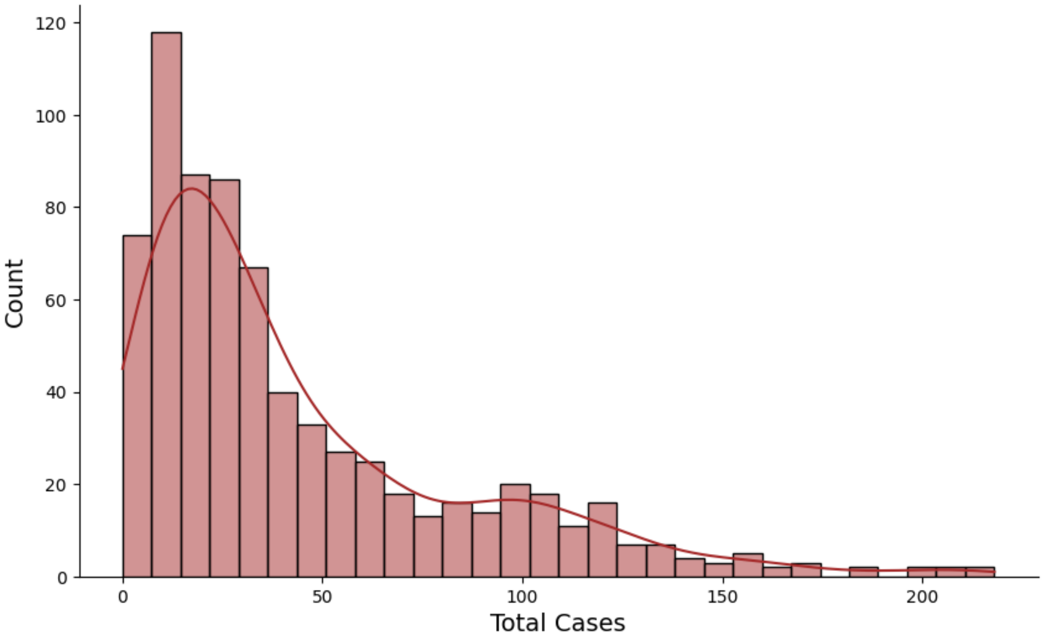


**Figure S3.** Distribution of the target variable (total cases)

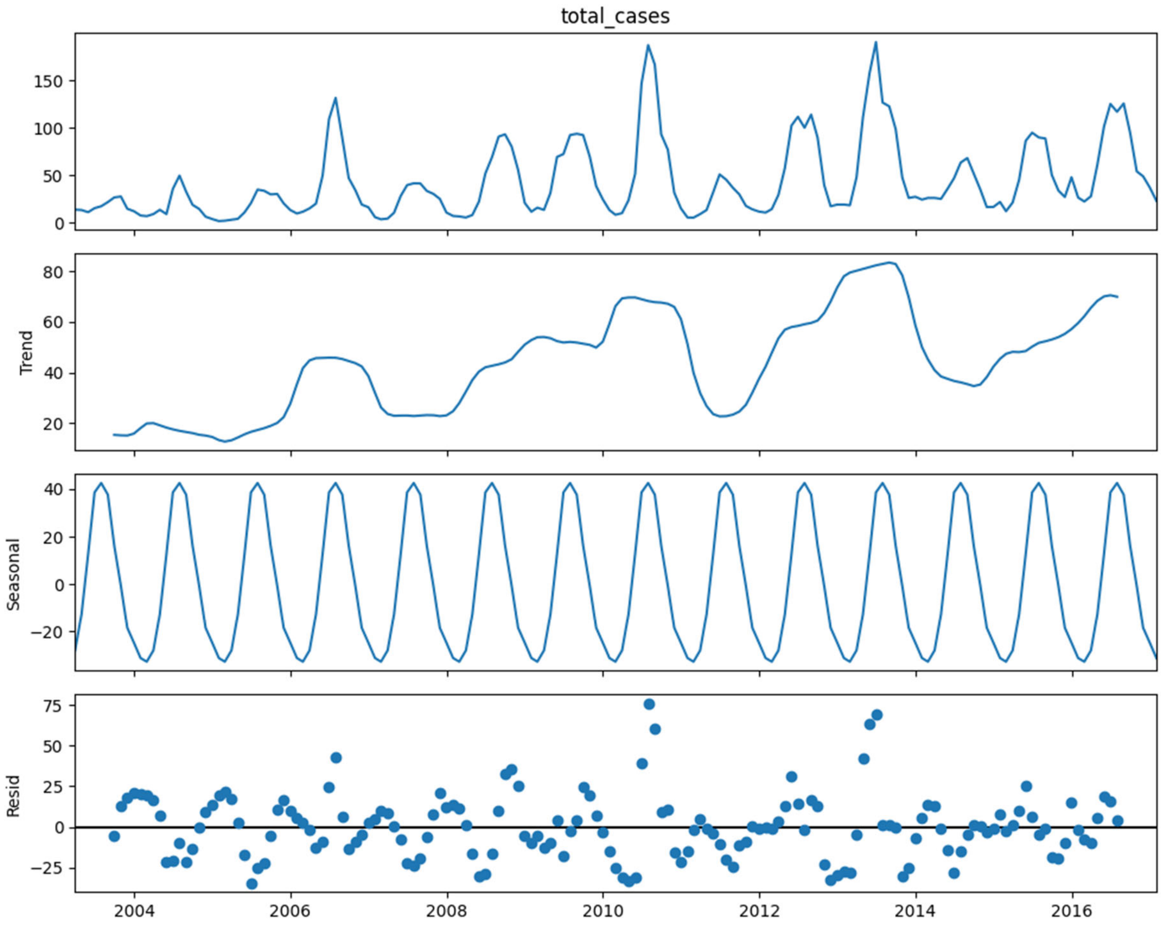


**Figure S4.** Seasonal and trend decomposition of the dengue case time series.

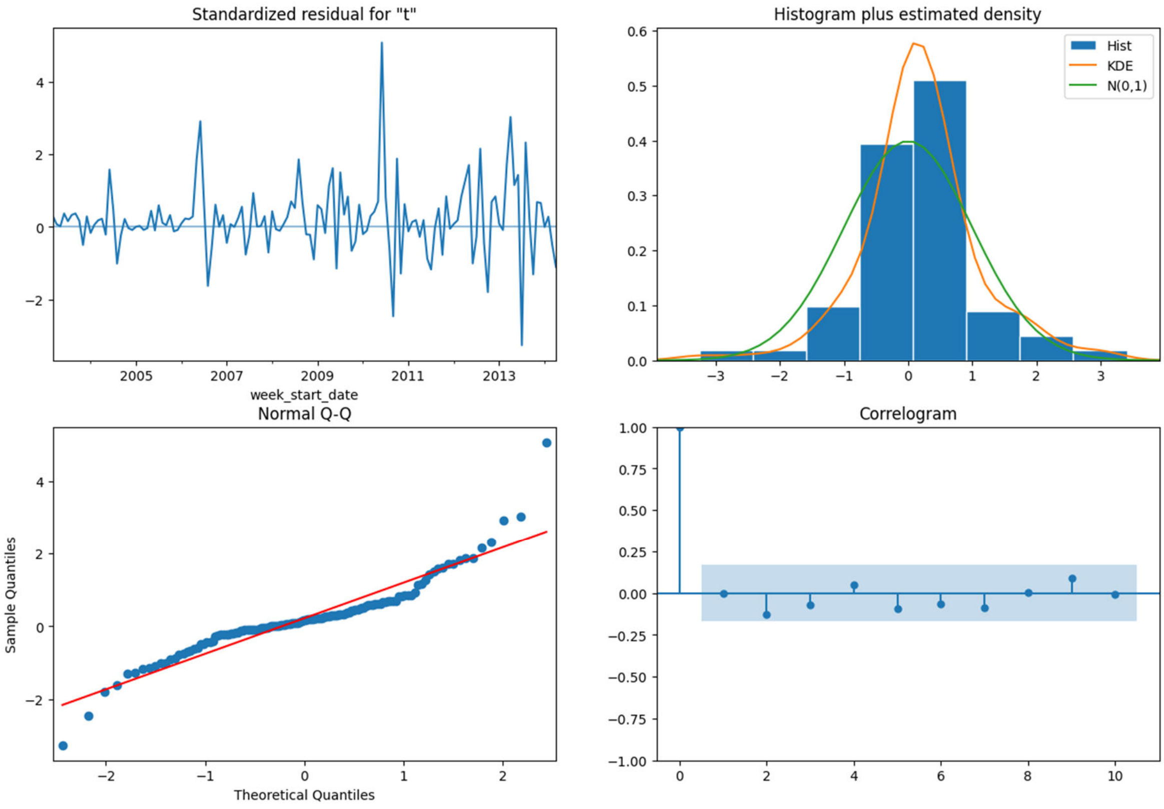


**Figure S5.** SARIMAX #1 Residual diagnostics.

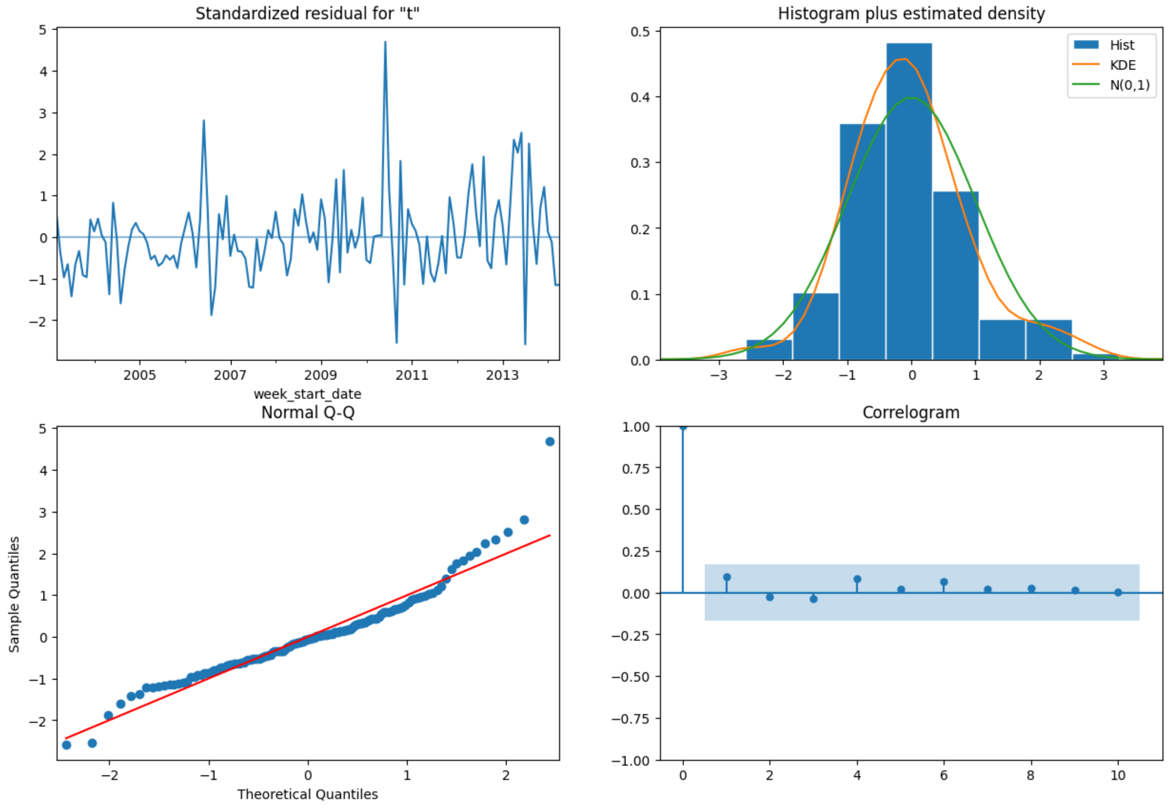


**Figure S6.** SARIMAX #2 Residual diagnostics.

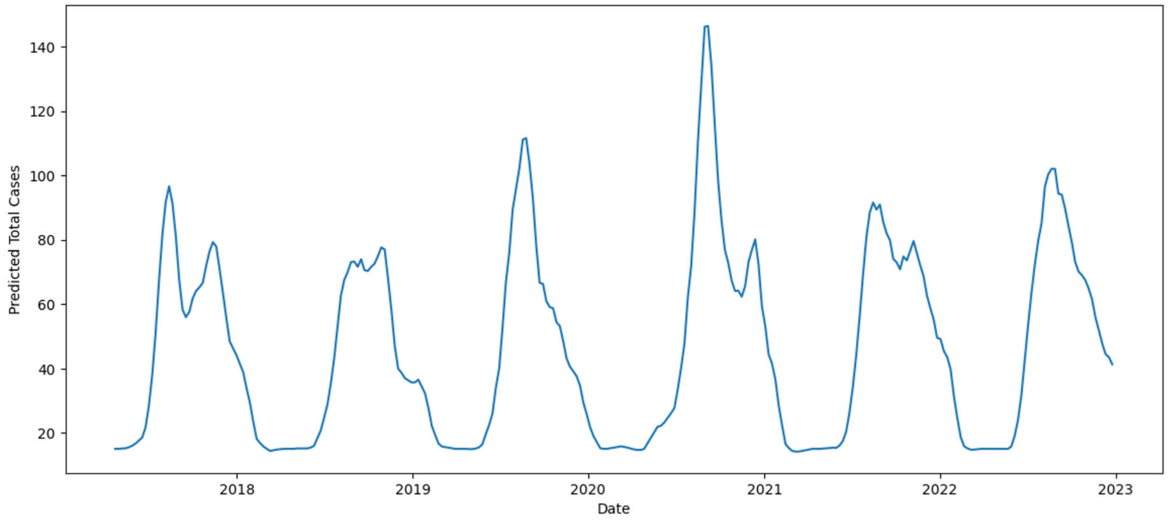


**Figure S7.** Zoomed-in forecast showing additional dengue peaks for XGB Regression Model #2.

**Table S1.** Parameter estimation and statistical diagnostics of SARIMAX #1.

**SARIMAX Results**

| | | | |
|---|---|---|---|
| **Dep. Variable:** | total_cases | No. Observations: | 134 |
| **Model:** | SARIMAX(2, 0, 0)x(1, 0, [1], 12) | Log Likelihood | -564.715 |
| **Date:** | Mon, 26 Feb 2024 | AIC | 1139.431 |
| **Time:** | 16:22:13 | BIC | 1153.92 |
| **Sample:** | 03/31/2003 | HQIC | 1145.319 |
| | -04/30/2014 | | |
| **Covariance Type:** | opg | | |

| | **coef** | **std err** | **z** | **P>\|z\|** | **[0.025** | **0.975]** |
|---|---|---|---|---|---|---|
| **ar.L1** | 1.2562 | 0.075 | 16.841 | 0 | 1.11 | 1.402 |
| **ar.L2** | -0.4348 | 0.06 | -7.226 | 0 | -0.553 | -0.317 |
| **ar.S.L12** | 0.9153 | 0.09 | 10.156 | 0 | 0.739 | 1.092 |
| **ma.S.L12** | -0.6794 | 0.135 | -5.036 | 0 | -0.944 | -0.415 |
| **sigma2** | 252.8124 | 24.472 | 10.331 | 0 | 204.849 | 300.776 |
| **Ljung-Box (L1) (Q):** | 0 | Jarque-Bera (JB): | 173.33 | | | |
| **Prob(Q):** | 1 | Prob(JB): | 0 | | | |
| **Heteroskedasticity (H):** | 3.15 | Skew: | 0.82 | | | |
| **Prob(H) (two-sided):** | 0 | Kurtosis: | 8.32 | | | |

**Table S2.** Parameter estimation and statistical diagnostics of SARIMAX #2.

| | **coef** | **std err** | **z** | **P>\|z\|** | **[0.025** | **0.975]** |
|---|---|---|---|---|---|---|
| **x1** | -0.1877 | 1.159 | -0.162 | 0.9 | -2.459 | 2.083 |
| **x2** | -25.9949 | 10.242 | -2.538 | 0 | -46.07 | -5.92 |
| **x3** | 4.4238 | 8.708 | 0.508 | 0.6 | -12.643 | 21.49 |
| **x4** | -1.2387 | 1.297 | -0.955 | 0.3 | -3.78 | 1.303 |
| **x5** | 1.5169 | 1.904 | 0.797 | 0.4 | -2.215 | 5.249 |
| **x6** | 0.8294 | 2.983 | 0.278 | 0.8 | -5.018 | 6.677 |
| **x7** | 0.1581 | 0.145 | 1.088 | 0.3 | -0.127 | 0.443 |
| **ar.L1** | 1.1303 | 0.072 | 15.723 | 0 | 0.989 | 1.271 |
| **ar.L2** | -0.4074 | 0.076 | -5.35 | 0 | -0.557 | -0.258 |
| **ar.S.L12** | 0.1794 | 0.101 | 1.775 | 0.1 | -0.019 | 0.378 |
| **sigma2** | 248.4918 | 25.431 | 9.771 | 0 | 198.648 | 298.336 |
| **Ljung-Box (L1) (Q):** | 1.22 | Jarque-Bera (JB): | 90.88 | | | |
| **Prob(Q):** | 0.27 | Prob(JB): | 0 | | | |
| **Heteroskedasticity (H):** | 2.07 | Skew: | 1.04 | | | |
| **Prob(H) (two-sided):** | 0.02 | Kurtosis: | 6.46 | | | |
